# Inhibitory leukocyte immunoglobulin-like receptors, subfamily B (LILRBs) in human diseases: structure, roles, mechanisms, and clinical applications

**DOI:** 10.7150/thno.116951

**Published:** 2025-07-25

**Authors:** Yuxiu Zhang, Yuanyuan Xu, Qihui Wu, Xiaodan Fu, Yimin Li, Anqi Li

**Affiliations:** 1 Department of Pathology, Ruijin Hospital, Shanghai Jiaotong University School of Medicine, Shanghai, China, 200025.; 2 Department of Pathology, Zhongshan Hospital, Fudan University, Shanghai, China, 200032.; 3 Department of Gynecology, Xiangya Hospital, Central South University, Changsha, China, 410008.; 4 National Clinical Research Center for Geriatric Disorders, Xiangya Hospital, Changsha, China, 410008.; 5 Department of Pathology, Xiangya Hospital, Central South University, Changsha, China, 410008.

**Keywords:** immunosuppressive receptors, hematological malignancies, solid tumors, immune regulation, immunotherapy

## Abstract

Leukocyte immunoglobulin-like receptors, subfamily B (LILRBs), are a class of critical immunosuppressive receptors that contribute to immune homeostasis by transmitting suppressive signals upon binding to ligands such as major histocompatibility complex class I molecules. They play key roles in modulating both innate and adaptive immune responses. This review summarizes the structural features, ligand interactions, signaling pathways, and expression regulation of LILRBs, and discusses their roles in immune cell function and disease progression, particularly in the tumor microenvironment. We also review current progress in the development of LILRB-targeted therapies for hematological malignancies and solid tumors and outline the challenges and future directions in translating these findings into clinical applications. By integrating recent advances, this review provides a framework for understanding the potential of LILRBs as therapeutic targets in cancer and immune-related disorders.

## 1. Introduction

The immune system maintains the ability to distinguish self from non-self through precise recognition and signaling mechanisms, enabling the clearance of pathogens while preserving host tissue integrity and immune homeostasis 1. However, dysregulation of this recognition process can trigger aberrant immune responses, leading to autoimmunity and chronic inflammation 1. From an immunological perspective, tumors can be viewed as non-self or altered self-entities. While the immune system is capable of recognizing and eliminating early-stage malignant cells, tumor cells often evade immune surveillance by expressing antigens shared with normal tissues or by downregulating antigen presentation machinery 1, 2. Additionally, they actively suppress immune cell functions and remodel the immune microenvironment to support immune evasion and tumor progression 1, 2.

In recent years, immunotherapy has revolutionized the treatment of infectious diseases, autoimmune conditions, and cancer by modulating immune responses with high precision 3. Core strategies, including monoclonal antibodies (mAbs), chimeric antigen receptor (CAR) T-cell therapy, therapeutic vaccines, and immune checkpoint blockade, have achieved notable clinical success 4-7. The remarkable efficacy of checkpoint blockade, in particular, has driven extensive research into its potential to mitigate autoimmune responses and inhibit tumor progression 8, 9. Nonetheless, challenges remain, including poor tissue penetration, off-target effects, and treatment-related toxicities, which limit the broader application of these therapies 10-13. Additionally, T-cell expansion and the abnormal elevation of inflammatory factors can lead to immune resistance or hypersensitivity reactions, further complicating therapeutic outcomes 14-16.

Inhibitory immune receptors serve as essential modulators that fine-tune immune activation and maintain immune homeostasis 17. Among these, the leukocyte immunoglobulin (Ig)-like receptors, subfamily B (LILRBs), represent a prominent class of inhibitory receptors with broad immunoregulatory roles. Accumulating evidence implicates LILRBs in the development and progression of infectious diseases, autoimmune disorders, and various malignancies 18-20. The human LILRB family consists of five members: LILRB1 (also known as LIR-1, ILT2, CD85j), LILRB2 (LIR-2, ILT4, CD85d), LILRB3 (LIR-3, ILT5, CD85a), LILRB4 (LIR-5, ILT3, CD85k), and LILRB5 (LIR-8, CD85c) 21. These receptors contain two to four cytoplasmic immunoreceptor tyrosine-based inhibitory motifs (ITIMs), which recruit Src homology 2 domain-containing phosphatases 1/2 (SHP1/2) to downregulate activating signaling pathways 18. LILRBs are predominantly expressed on myeloid lineage cells, including monocytes, macrophages, neutrophils, and dendritic cells (DCs), as well as certain tumor cells 18, 20, 21. LILRB1 represents the exception, with additional expression documented on lymphocytes such as B cells and specific subsets of natural killer (NK) cells and T cells 18, 20, 21. In mice, paired Ig-like receptor B (PirB) is considered the functional ortholog of human LILRB2/3, while Lilrb4a (also known as gp49b) shares homology with LILRB4 20. These murine receptors have been widely used in preclinical studies to investigate LILRB functions in immune regulation and disease pathology. Emerging evidence has revealed aberrant expression of LILRBs across multiple tumor types, where they regulate diverse oncogenic processes, including tumor proliferation, invasion, metastasis, and immune evasion 22-25. These findings highlight LILRBs as potential diagnostic biomarkers and therapeutic targets in oncology, warranting further translational investigation.

This review systematically summarizes the structure features, ligand interactions, signaling mechanisms, and regulatory pathways governing LILRB expression. We further explore their expression and functional implications in the immune system and diseases, with particular emphasis on their impact on tumor cells and the tumor microenvironment (TME). Additionally, the review compiles information on clinical trial drugs targeting LILRBs, covering drug types, mechanisms of action, and trial stages. By integrating recent advances, this review aims to provide a theoretical foundation for the development of novel LILRB-targeted therapies and to foster innovation in disease treatment strategies.

## 2. Structure, Ligands, and Signaling Pathways of LILRBs

### 2.1 Structure of LILRBs

**Figure 1:**
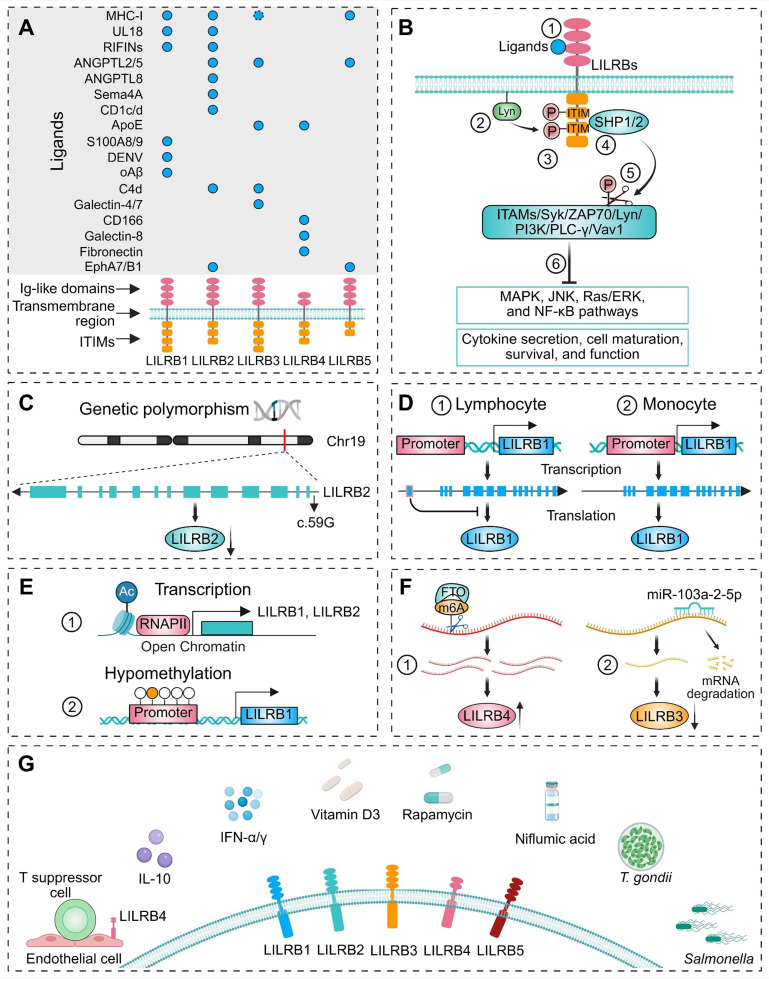
** Structure, ligands, signal transduction pathways, and upstream regulatory mechanisms of LILRBs.** (A) Top: Bubble chart displaying ligands of LILRB1-5, ordered by their introduction sequence in text, with columns representing corresponding LILRB receptors. Bottom: Domain architecture of individual LILRB receptors: extracellular Ig-like domains (red), transmembrane regions, and intracellular ITIMs (yellow). The dashed circle indicates that the functional role of this ligand-receptor interaction remains controversial. (B) Canonical LILRB-mediated downstream signaling pathway: ① Ligand binding to LILRBs triggers ② Lyn kinase autophosphorylation; ③ phosphorylates ITIM motifs in intracellular domains, leads to ④ recruitment and activation of SHP1/2, which ⑤ dephosphorylate downstream signaling proteins, ultimately ⑥ modulating downstream signaling cascades and cellular functions. (C) A polymorphism at c.59G in the 5'-UTR region modulates LILRB2 expression. (D) Differential promoter usage of LILRB1 in ① lymphocyte versus ② monocyte results in an additional exon in lymphocyte that suppresses protein translation. (E) Transcriptional regulation of LILRB1 and LILRB2 by epigenetic modifications, including ① histone acetylation and ② DNA methylation in promoter regions. (F) Post-translational regulatory mechanisms: ① FTO enhances LILRB4 expression via m6A demethylation, ② miR-103a-2-5p suppresses LILRB3 expression through mRNA degradation. (G) LILRB expression modulation by intercellular crosstalk, cytokines, pharmacological agents, and microbial infections.

LILRB family consist of inhibitory transmembrane glycoproteins characterized by extracellular Ig-like domains, a single transmembrane segment, and multiple intracellular ITIMs. These receptors are encoded within the leukocyte receptor complex gene cluster on chromosome 19q13.4 and were first cloned in 1997 26-29. LILRB1-3 and LILRB5 share a conserved domain architecture consisting of four extracellular Ig-like domains (D1-D4), whereas LILRB4 contains only two such domains (Figure 1A) 21. Structural analyses have revealed distinct interdomain geometries in LILRB1 and LILRB2, especially at the D2-D3 hinge (LILRB1: ~60°; LILRB2: ~50°), a difference attributed to a single amino acid substitution (W284 in LILRB1 vs. C283 in LILRB2) that affects hydrophobic packing and steric compatibility 30. While the D3-D4 domains acted as a rigid scaffold without direct human leukocyte antigen (HLA)-G1 contact, the staggered assembly of both receptors restricted interdomain flexibility, confining ligand recognition exclusively to D1 and D2 30, 31. Specifically, the D1 domain engaged the α3 domain of HLA-G1, and the D2 domain interacted primarily with β₂-microglobulin (β2m), forming a conserved binding interface essential for HLA class I (HLA-I) recognition. Notably, the D1 and D2 domains exhibited significant conformational plasticity, enabling adaptation to diverse HLA ligands (e.g., HLA-A2, HLA-F, UL18) 32, 33, which facilitates robust immune regulation across varied HLA polymorphisms and pathogenic contexts. LILRB4 adopted a similar overall fold but displayed unique structural features. Its D2 domain most closely resembled the D4 domains of other LILRB members and uniquely contains 3₁₀-helices 34. The reduced interdomain contacted between D1 and D2 result in a wider interdomain angle (~107°), producing a more open conformation 34. Due to both conformational and electrostatic incompatibility, LILRB4 is poorly suited for MHC class I (MHC I) binding. Instead, distinct surface patches on its D1 domain and hinge region suggested interaction with non-MHC-I ligands 34.

Anchored by a specific transmembrane region, LILRBs connect their extracellular ligand-binding domains to cytoplasmic ITIM-containing tails (Figure 1A). ITIMs are a class of conserved amino acid sequences located in the cytoplasmic tails of certain transmembrane receptors (Figure 1A), characterized by unique structural features. The canonical ITIM sequence is S/I/V/LxYxxI/V/L, where "x" denotes any amino acid 35. Although the intracellular tails of LILRBs lack typical kinase domains, they exert inhibitory signaling by recruiting phosphatases such as SHP1 and SHP2 upon phosphorylation, thereby modulating immune responses.

### 2.2 Ligands of LILRBs

#### MHC-I molecules

MHC-I molecules play a pivotal role in immune surveillance by presenting intracellular peptides to T cells and NK cells 36. These molecules are typically expressed on the surface of nucleated cells and consist of a trimeric complex formed by heavy chains (α1-α3) and an invariant light chain, β2m. The heavy chains span the cell membrane, while the domains distant from the membrane, including α1 and α2, form the peptide-binding groove 37. The heavy chains of MHC-I molecules in human are part of the HLA system. Classical MHC-I molecules are encoded by classical MHC-I genes: HLA-A, HLA-B, and HLA-C, while non-classical MHC class I genes include HLA-E, HLA-F, and HLA-G 37. β2m binds non-covalently to the extracellular portion of the heavy chain but does not directly interact with the cell membrane 38. LILRB1 and LILRB2 transmit inhibitory immune signals by interacting with the α3 domain and the β2m subunit of classical MHC-I molecules (e.g., HLA-A and HLA-B) on the surface of target cells 38-40. MHC-I molecules exist in two forms on the cell surface: the β2m-associated form and a free heavy chain with an open conformation 41. Notably, LILRB1 specifically recognizes the β2m-associated form and fails to bind to MHC-I molecules lacking β2m 42. In contrast, LILRB2 has broader ligand recognition and can interact with β2m-free heavy chains, such as those of HLA-B27 43, HLA-C 44 and HLA-G 42, 45. In contrast, the interaction between LILRB3 and MHC-I remains controversial. Several studies have reported that MHC-I molecules do not bind to LILRB3 43, 44, 46, or that HLA-G fails to induce the conformational changes necessary for LILRB3 activation in myeloid cells, as shown in a recent study 47. However, Ayukawa et al. demonstrated that LILRB3 on healthy epithelial cells binds to the α3 domain of MHC-I on transformed cells, triggering mechanical force via the SHP2/Rho-associated protein kinase 2 (ROCK2) pathway to promote extrusion and apoptosis of precancerous cells 48. These findings suggest that the binding affinity and functional consequences of the LILRB3-MHC-I interaction are likely to be highly context-dependent, varying according to cell type and microenvironmental cues. LILRB5 has also been shown to bind to certain classical MHC-I molecules. Zhang et al. reported that the heavy chains of HLA-B7 and HLA-B27 can bind to LILRB5 in transduced B and rat basophil RBL cell lines 49.

Similar to classical HLA-I molecules, HLA-G comprises a heavy chain, β2m, and an 8-10 amino acid peptide derived from intracellular proteolytic degradation products 42. However, it binds to LILRB1 and LILRB2 with greater affinity than classical MHC-I molecules 42, 45. Through these interactions, HLA-G can inhibit cytotoxic T cells, NK cells, and B cells, induce T-cell anergy, regulate myeloid cell functions, and promote the generation and activation of regulatory T cells (Tregs) 50. HLA-G exists in multiple membrane-bound and soluble isoforms, generated through alternative splicing or proteolytic cleavage 41. Notably, HLA-G can form disulfide-linked homodimers, which are more effective than monomers at engaging LILRBs and transmitting inhibitory signals 51-54. HLA-F, one of the least characterized non-classical MHC-I molecules, is primarily retained intracellularly in resting leukocytes. However, its surface expression has been observed in B lymphoblastoid and monocyte-derived cell lines 55. Surface plasmon resonance studies have shown that tetrameric complexes of HLA-F can directly interact with LILRB1 and LILRB2 56.

#### Membrane glycoprotein UL18 (UL18)

The human cytomegalovirus (CMV) UL18 gene encodes a highly glycosylated transmembrane protein that shares approximately 25% sequence homology with host MHC-I molecules 57. UL18 is proposed to act as a viral decoy, mimicking MHC-I structure to engage killer-cell immunoglobulin-like receptors (KIRs) on NK cells and thereby inhibit NK cell-mediated cytotoxicity 58. Similar to host MHC-I molecules, UL18 can bind β2m and endogenous peptides 28. Structurally, UL18 contains α1, α2, and α3 domains in its extracellular region, with the α3 domain primarily interacting with the D1 domain of LILRB1 59. Although the binding mode of LILRB1 to UL18 resembles its interaction with classical MHC-I molecules, LILRB1 exhibits an affinity for UL18 that is over 1,000-fold higher than for either classical or non-classical MHC-I ligands 59, 60. In addition to the binding sites for LILRB1 and the host-derived light chain, crystal structure analyses revealed that UL18 is extensively shielded by carbohydrate moieties, which prevent interactions with many potential binding partners 60. Such structural adaptations enabled UL18 to outcompete host MHC molecules for LILRB1 binding, thereby disrupting immune surveillance mechanisms 60. Despite the high sequence homology among LILRB family members, only LILRB1, and to a lesser extent LILRB2, can bind UL18 26, 33, 61.

#### Repetitive interspersed families of polypeptides (RIFINs)

RIFINs are variant antigens encoded by the *rif* gene families in *Plasmodium falciparum* (*P. falciparum*) and expressed on the surface of infected red blood cells 62. These proteins contribute to immune evasion by binding to LILRB1, thereby inhibiting the activation of B cells and NK cells 63. The extracellular domain of RIFINs comprises a relatively conserved N-terminal region, which includes the FHEYDER sequence, and a highly variable C-terminal region 63. Although RIFINs share no sequence or structural homology with HLA-I molecules, Harrison et al. demonstrated that their variable regions can interact with the D1 and D2 domains of LILRB1, mimicking the binding pattern of classical MHC-I molecules 64. Further structural analyses by Chen et al. revealed that RIFINs also directly engage the D3 domain of LILRB1 via specific amino acid residues or conformational features within their variable regions 65. These findings suggest that the polygenicity and polymorphism of the RIFIN family reflect strong selective pressure to evade the antibody response while evolving multiple mechanisms to engage inhibitory receptors 65. Although LILRB2 shares significant structural similarity with LILRB1, exhibiting 81% amino acid homology in the extracellular region 33, 42, functional assays indicate a distinct binding pattern. Blocking assays with anti-LILRB2 antibodies and binding assays with recombinant LILRB2 proteins have shown that RIFINs primarily interact with the D3 domain of LILRB2, rather than D1 or D2 domain 66. Elucidating the interactions between RIFINs and LILRB1/LILRB2 not only sheds light on the immune evasion strategies of *P. falciparum* but also provide novel insights for the development of malaria treatments and vaccines.

#### Angiopoietin-like proteins (ANGPTLs)

ANGPTLs are a class of proteins structurally similar to angiopoietins and play a significant role in regulating angiogenesis 67. Unlike angiopoietins, ANGPTLs do not bind to the angiopoietin receptors Tie1 or Tie2, which led to their initial classification as "orphan ligands" 68, 69. In 2012, Zheng et al. identified LILRB2 as the receptor for ANGPTL2 and ANGPTL5, demonstrating that signaling through this receptor maintains hematopoietic stem cell (HSC) stemness and promotes leukemia development 70. A subsequent study identified that the HGY*C motifs within the D1 and D4 domains of LILRB2 are essential for binding ANGPTL2 and activating downstream signaling pathways 71. Notably, the interaction between LILRB2 and ANGPTL2 exhibited a higher binding affinity than that with HLA-G, with partially overlapping yet distinct binding sites on LILRB2 71. Although ANGPTL2 and ANGPTL5 also interacted with LILRB3 and LILRB5, the binding affinity was lower than that of ANGPTL2 and LILRB2 72. ANGPTL8 is predominantly expressed in human liver 73 and is closely associated with non-alcoholic fatty liver disease in both mice and humans 74. ANGPTL8 bound its receptor PirB/LILRB2 to regulate macrophage migration in nonalcoholic steatohepatitis (NASH), and its hepatocyte-specific deletion reduced macrophage infiltration, lipid accumulation, and fibrosis progression in NASH mice 75.

#### Semaphorin-4A (Sema4A)

Sema4A was initially identified in developing embryos, with its transcriptional levels progressively increasing throughout embryonic development 76. This protein plays critical roles in neural development and immune regulation 77. Lu et al. demonstrated that human SEMA4A co-stimulates CD4+ T cells and regulates T helper 2 (Th2) cell differentiation by binding to LILRB2 on activated CD4+ T cells 77.

#### CD1 family

The human CD1 family consists of four functional subtypes: CD1a, CD1b, CD1c, and CD1d. These glycoproteins are expressed on the cell surface and function as the third class of antigen-presenting molecules 78. Structurally, CD1 molecules resemble MHC-I molecules, notably through the non-covalent association of their heavy chains with β2m 79, 80. LILRB2 functions as an inhibitory receptor for CD1c and CD1d, suppressing immune responses triggered by these molecules 80, 81.

#### Apolipoprotein E (ApoE)

ApoE is a plasma protein that acts as a ligand for the low-density lipoprotein (LDL) receptor, facilitating the transport of cholesterol and other lipids between diverse cell types 82. In terms of immune regulation, studies have presented conflicting conclusions regarding the relationship between ApoE and immune receptors. One study demonstrated that among ApoE isoforms, ApoE4 can bind a variant of LILRB3 and activate human microglia HMC3 cells in a LILRB3-dependent manner, promoting a proinflammatory state 83. However, Huang et al. reported that no ApoE isoform, including ApoE4, activate LILRB3 reporter cells 47. In contrast, the same study found that all ApoE isoforms could activate LILRB4 reporter cells, consistent with prior research 22, 47, 84. Given these discrepancies, Huang et al. proposed that while ApoE may bind LILRB3 with measurable affinity, this interaction—under their experimental conditions—failed to induce the functional conformational changes necessary for LILRB3 activation 47.

#### Other ligands

Several studies have demonstrated that LILRB1 can bind not only to the calcium-binding proteins S100 calcium binding protein A8 (S100A8) and S100A9 85, but also to products of the Dengue virus (DENV) 86. In a mouse model of Alzheimer's disease (AD), oligomeric β-amyloid (Aβ) interacted with LILRB2, leading to synaptic loss 87. Additionally, LILRB2 and LILRB3 can interact with soluble monomeric complement component C4d, mediating its endocytosis 88. Using a LILRB3 reporter cell system, Huang et al. demonstrated that galectin-4 and galectin-7 can activate LILRB3 and induce the downstream SHP1/2 signaling 47. LILRB4 binds ligands including activated leukocyte cell adhesion molecule (ALCAM)/CD166 89, galectin-8 90, and fibronectin 91, 92, regulating downstream cellular activities. A recent study demonstrated that LILRB5 (and partially LILRB2) forms specific interactions with Eph receptors (EphA7 and EphB1), triggering bidirectional signaling through phosphorylation of both ITIM domains in LILRB5 and Eph receptors 93. This crosstalk induced an immunosuppressive phenotype in myeloid cells, and suppresses antitumor T cell responses, therefore supporting tumor progression.

### 2.3 LILRBs and signal transduction pathways

LILRB receptors interact with various ligands, including MHC-I molecules and other immune-related ligands, initiating inhibitory signal transduction through ITIMs within their cytoplasmic domains 18, 23, 94, 95. The Src family kinases, particularly Lck/Yes-related novel protein tyrosine kinase (Lyn), play a central role in LILRB signaling 96, 97. Upon receptor engagement, Lyn undergoes autophosphorylation and subsequently phosphorylates the tyrosine residues within the ITIMs (Figure 1B) 20. These phosphorylated residues serve as docking sites for critical negative regulators SHP1/2 98, which, upon recruitment, dephosphorylate key signaling molecules 99, 100. These molecules include immunoreceptor tyrosine-based activation motifs (ITAMs), Src family kinases, spleen tyrosine kinase (Syk), zeta-chain-associated protein kinase 70 kDa (ZAP70), Lyn, phosphatidylinositol-4-phosphate 3-kinase (PI3K), phospholipase C gamma (PLC-γ), and Vav guanine nucleotide exchange factor 1 (Vav1) (Figure 1B) 97, 101-104. This dephosphorylation inhibits downstream signaling pathways, such as mitogen-activated protein kinase (MAPK), c-Jun N-terminal kinase (JNK), Ras/extracellular signal-regulated kinase (ERK), and NF-κB pathways, ultimately suppressing cytokine secretion, effector cell maturation, survival, and function (Figure 1B) 97, 98. The following sections will delve into the interactions between LILRBs, their ligands, and downstream signaling molecules in specific diseases and cell types.

## 3. Upstream Modulators of LILRBs

Recent studies have demonstrated that polymorphisms in LILRBs significantly contribute to the pathogenesis and progression of multiple diseases 32, 94, 105-111. Polymorphisms can alter gene sequences, affecting transcription factor binding, mRNA processing, and translation, thereby modulating gene expression levels. Additionally, polymorphisms in coding regions may lead to amino acid substitutions that disrupt protein folding and structural stability. Notably, these disease-associated polymorphisms exhibit distinct ethnic distributions 110, 112-114. For instance, Hirayasu et al. identified LILRB2 polymorphisms within 5'-UTR and signal sequence regions in northeast Asian populations that directly influence its expression (Figure 1C) 113. Flow cytometry confirmed the association between the common LILRB2 c.59G allele and its lower expression 113. In Brazilian populations, next-generation sequencing identified six amino acid substitutions in LILRB1 and five in LILRB2 114. These allelic variations may affect the structure and/or stability of these molecules, with LILRB2 demonstrating higher average stability. Collectively, these findings provide critical insights into population-specific functional diversification of LILRBs. LILRBs are most highly expressed in myeloid hematopoietic cells, with relatively lower expression in non-hematopoietic tissues, though the precise mechanisms underlying these patterns remain incompletely understood 18, 21. LILRB expression is regulated at multiple levels, including transcriptional, epigenetic, and post-transcriptional mechanisms, each contributing to the cell type-specific expression profiles of these receptors. For instance, LILRB1 expression was controlled by cell-specific promoters: a lymphocyte-specific promoter located approximately 13 kb upstream of the monocyte-specific promoter introduced a unique exon into the 5'-UTR, which inhibited protein translation, resulting in lower protein levels in lymphocytes compared to monocytes (Figure 1D) 115. In multiple myeloma (MM), the transcription factor Ikaros family zinc finger protein 1 (IKZF1) directly activated LILRB4 transcription 116. Beyond transcriptional regulation, epigenetic modifications also play a crucial role in controlling LILRB expression by modulating chromatin accessibility at gene promoters. In addition to transcriptional regulation, epigenetic modifications further fine-tune LILRB expression. These modifications, such as histone acetylation and DNA methylation, contribute to gene expression by altering chromatin structure and accessibility. The promoters of LILRB1 and LILRB2 share several cis-elements and trans-factors, but their activation mechanisms differ. LILRB1 transcription relies on Sp1-family binding to its GC-box, while LILRB2 is tightly regulated by histone acetylation at its core promoter, which restricts its expression to myeloid lineage cells (Figure 1E) 117. Another critical epigenetic modification is DNA methylation, with TCGA analysis revealing a significant association between LILRB1 promoter hypomethylation and its elevated expression in low-grade glioma and glioblastoma (Figure 1E) 118. Post-transcriptional mechanisms, including RNA modifications and microRNA-mediated regulation, also contribute to the fine-tuning of LILRB expression. For instance, the N6-methyladenosine (m6A) demethylase FTO stabilized LILRB4 mRNA by removing m6A modifications, thereby promoting LILRB4 expression and contributing to leukemia stem cell self-renewal and immune evasion (Figure 1F) 119. Similarly, in acute myeloid leukemia (AML), overexpression of miR-103a-2-5p suppressed LILRB3 expression, inhibiting AML cell growth and reducing apoptosis in CD8+ T cells (Figure 1F) 120.

LILRB expression is dynamically regulated through cell-cell interactions, immune signaling, and exogenous factors (Figure 1G). Alloantigen-specific CD8+CD28- T suppressor cells upregulated LILRB4 in endothelial cells to promote immune tolerance (Figure 1G) 121. Mechanistic studies revealed that in quiescent endothelial cells, LILRB4 pre-mRNA is retained in the nucleus. Upon interaction with T suppressor cells or exposure to interleukin-10 (IL-10) and interferon-alpha (IFN-α), LILRB4 pre-mRNA processing was activated, leading to LILRB4 protein production 122. Beyond cell-cell interactions, immune signals further modulate LILRB expression via inflammatory stimuli, cytokines, and growth factors (Figure 1G) 123. In DCs, IL-10 124 and IFN-γ 125, 126 have been shown to enhance LILRB expression, as well as pharmacological agents such as vitamin D3 126, 127, rapamycin 128, and certain non-steroidal anti-inflammatory drugs (e.g., niflumic acid) 129 can also upregulate LILRB expression (Figure 1G). Microbial infections, including *Toxoplasma gondii* (*T. gondii*) 130 and *Salmonella* 131, also regulate LILRB expression (Figure 1G). During pregnancy, *T. gondii* infection downregulated macrophage LILRB4, driving proinflammatory M1 polarization while suppressing M2-type immunoregulatory tolerance 130, 132, 133. This shift altered M1/M2 surface markers, dysregulated arginine metabolic enzymes, and modified cytokine secretion profiles, ultimately disrupting fetal-maternal tolerance and contributing to adverse outcomes including preterm birth, miscarriage, and anencephaly 130.

These findings collectively delineate the multifaceted regulatory landscape of LILRB expression, spanning genetic polymorphisms, post-transcriptional modifications, immune cell interactions, and environmental stimuli. Such intricate regulatory networks underscore the pivotal role of LILRBs in modulating immune homeostasis, providing mechanistic insights into immune evasion, immune tolerance, and related biological processes. This refined understanding advances fundamental knowledge of immune regulation and elucidates the mechanisms underlying immune-related disorders, highlighting LILRBs as potential therapeutic targets.

## 4. LILRBs and Immune System

LILRBs constitute a class of inhibitory receptors broadly expressed on immune cells—including monocytes, macrophages, NK cells, T cells, and B cells 134—that critically modulate both innate and adaptive immunity 18, 27, 135. Emerging evidence reveals their dual role in regulating antigen-presenting cell (APC) functions while simultaneously suppressing cytotoxic anti-tumor responses and actively remodeling the TME 21. Notably, certain LILRBs are also expressed on tumor cells, where they directly influence cancer initiation, progression, therapeutic resistance, recurrence, and cancer stem cell behavior 97. This section explores LILRB-mediated immunomodulation, with a particular focus on their mechanisms of action within the TME.

### 4.1 Innate immunity

HSCs in the bone marrow (BM) serve as the progenitors of immature myeloid cells (IMCs), which subsequently differentiate into monocytes—capable of further maturing into macrophages and DCs—as well as granulocytes, including neutrophils, basophils, and eosinophils 136. Innate immune cells, encompassing myeloid-derived cells (e.g., monocytes, DCs, and macrophages) and innate lymphoid cells such as NK cells, critically mediate innate immunity and orchestrate inflammatory responses 137-139.

**Figure 2:**
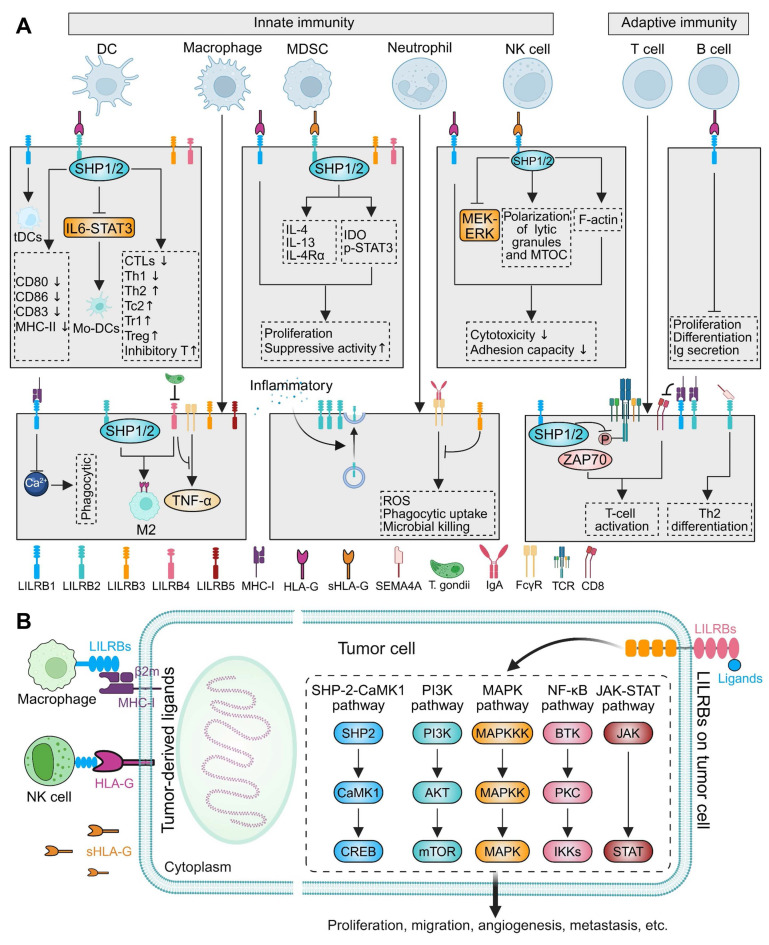
** Roles of LILRBs in innate immunity, adaptive immunity, and tumor-immune crosstalk.** (A) Distribution of LILRBs across innate immune cells (left; including dendritic cells (DCs), macrophages, myeloid-derived suppressor cells (MDSCs), neutrophils, and natural killer (NK) cells) and adaptive immune cells (right; including T and B lymphocytes). The diagram highlights associated ligands, signaling pathways, and immunomodulatory outcomes. (B) LILRB-mediated tumor-immune crosstalk. The left panel depicts tumor cell-derived ligands engaging LILRB-expressing immune cells, modulating anti-tumor responses. The right panel illustrates how tumor cells, upon stimulation by exogenous ligands, activate intracellular signaling pathways (e.g., SHP2/CaMK1, PI3K/AKT, MAPK, NF-κB, and JAK/STAT) to regulate tumor progression and immune evasion.

DCs serve as pivotal APCs bridging innate and adaptive immunity, initiating immune responses while maintaining immune tolerance 140, 141. DCs exist in various subsets and functional states, with immature forms primarily involved in establishing immune tolerance and mature forms critical for inducing protective T-cell-mediated immunity 142. LILRB expression on DCs is dynamically regulated by inflammatory stimuli, cytokines, and growth factors, and is typically downregulated upon DC activation 123. For instance, HLA-G binding to LILRB2 suppressed DC activation and downregulated co-stimulatory molecules (CD80, CD86, CD83, and MHC class II) (Figure 2A) 143, recruiting SHP1/2 to inhibit monocyte-to-DC differentiation via the IL-6/STAT3 signaling pathway (Figure 2A) 144. Similarly, LILRB1 and LILRB4 were downregulated upon DC activation, suggesting their involvement in regulating DC maturation 123. Persistent LILRB1 ligation during monocyte culture generated tolerogenic DCs (tDCs), which interacts with Tregs and suppresses T-cell responses 145. Complementary to this, LILRB2+ DCs inhibited the differentiation of T helper 1 (Th1) cells and cytotoxic T lymphocytes (CTLs), while promoting the generation of Th2 and type 2 cytokine-secreting CD8+ T cells (Figure 2A) 146-148. Additionally, LILRB2 contributed to the induction of various Treg subsets, including CD4+CD25+ Tregs, CD8+CD28- inhibitory T cells, and type 1 Tregs, which are critical for maintaining immune tolerance to both self- and non-self-antigens (Figure 2A) 149-151. In the dermis, CD14+ DCs expressing LILRB1 and LILRB2 exhibited a reduced capacity to induce cytotoxic CD8+ T cells, compared to LILRB-negative Langerhans cells, and blockade of LILRB1 and LILRB2 significantly enhanced CTL generation 148.

Macrophages, another pivotal mediator of innate and adaptive immunity, primarily derive from circulating monocytes and polarize into M1 or M2 subtypes in response to various stimuli 152, 153. M1 macrophages are closely associated with inflammatory responses, while M2 macrophages are primarily involved in anti-inflammatory processes, tissue repair and immunoregulation 153. The M2 spectrum includes tumor-associated macrophages (TAMs/M2d), which drive tumor progression, angiogenesis, and metastasis 153. LILRBs critically regulate macrophage function by modulating polarization, phagocytic capacity, and intercellular signaling. In TAMs, LILRB1 and LILRB2 are significantly upregulated, while blocking these receptors enhances macrophage phagocytosis (Figure 2A) 154, 155. LILRB1 inhibition may disrupt calcium mobilization following MHC-I engagement, impairing macrophage activation 27. LILRB2 promotes the polarization of TAMs toward M2-like phenotypes, which are associated with immune suppression and tumor progression (Figure 2A). In contrast, LILRB2 blockade repolarized TAMs to M1-like phenotypes by suppressing SHP1/2 phosphorylation and activating the NF-κB pathway, thereby augmenting anti-tumor immunity 155, 156. LILRB3, predominantly expressed on myeloid cells, especially monocytes, when agonistically bound by monoclonal antibodies to Ig-like domains 2 or 4, suppresses M1-inflammatory pathways and induces an M2-like immunosuppressive phenotype, leading to reduced T cell proliferation and immune suppression 157. LILRB4 and LILRB5 are also expressed in macrophages 21, 99, 158. In a midgestation murine model, macrophage-expressed gp49B (LILRB4 homolog) colligated with FcγRI to suppress tumor necrosis factor (TNF)-α production, maintaining maternal-fetal immune tolerance (Figure 2A) 159. During *T. gondii* infection, LILRB4 downregulation on decidual macrophages promoted M1 polarization and impaired M2-mediated tolerance functions (Figure 2A) 130. Conversely, NLRP12-induced LILRB4 upregulation in TAMs promoted M2 polarization within tumors 160.

Myeloid-derived suppressor cells (MDSCs) are a population of cells derived from IMCs under conditions of chronic inflammation, cancer, and autoimmune diseases. In these pathological states, persistent inflammatory signals disrupt their normal differentiation pathways and induce pathological activation. MDSCs exhibit an immature phenotype, reduced phagocytic activity, and potent immunosuppressive functions, enabling them to effectively suppress immune responses 136. HLA-G has been proven to promote MDSC expansion and suppressive function through its interaction with LILRB1, which increases the secretion of IL-4 and IL-13 and upregulates IL-4Rα, leading to T cell proliferation inhibition (Figure 2A) 161. Additionally, soluble HLA-G (sHLA-G) binding to LILRB2 on granulocytic MDSCs, enhancing indoleamine-2,3-dioxygenase (IDO) expression and promoting STAT3 phosphorylation, thus facilitating MDSC accumulation and their immunosuppressive activity (Figure 2A) 162. LILRB3 is functionally expressed on immunosuppressive myeloid cells from cancer patients, and targeted inhibition of this receptor has been shown to reverse MDSC-mediated immunosuppression 47, 163. Furthermore, LILRB4 expression on monocytic MDSCs plays a crucial role in mediating cancer-related immunosuppression, and its blockade can reverse this suppression, thereby enhancing the efficacy of immune checkpoint inhibitors (ICIs) 164.

Neutrophils play activating, regulatory, and effector roles in both innate and adaptive immunity 165, 166. As the most abundant granulocytes, their activation and function are finely regulated by LILRBs 20. Neutrophil granules contain various membrane receptors, which can be translocated to cell surface through exocytosis 167. In response to inflammatory stimulation, LILRB2 expression on the neutrophil surface was rapidly upregulated via granule exocytosis, thereby enhancing HLA-G-mediated suppression of neutrophil phagocytic function (Figure 2A) 167. Additionally, LILRB3 is highly expressed on resting neutrophils and is shed following activation 168, 169. Sustained LILRB3 ligation inhibited key IgA-mediated effector functions, including reactive oxygen species (ROS) production, phagocytosis, and microbial killing (Figure 2A) 168, 169.

NK cells defend against infections and tumors through direct lysis of target cells and the secretion of immune mediators 170. The effector functions of NK cells are regulated by MHC-I molecules, whereby target cells expressing MHC-I exhibit heightened resistance to NK-mediated killing relative to MHC-I-deficient cells 171. This regulatory mechanism is mediated by the interaction between HLA-I molecules and inhibitory NK receptors 172. Upon ligand binding, these inhibitory receptors undergo tyrosine phosphorylation at their intracellular ITIM domains, recruiting the tyrosine phosphatase SHP1, which suppresses the effector functions driven by activating receptors on NK cells 171. Conversely, in the absence of inhibitory signals, the engagement of activating receptors triggers target cell lysis and cytokine secretion 171. LILRB1 inhibited NK cell cytotoxicity by binding HLA-G1, and its blockade conferred partial protection against NK cells (Figure 2A) 173-175. In vitro, soluble HLA-G1α binding to LILRB1 inhibited downstream signaling molecules, including MEK and ERK, thereby suppressed NK cell cytotoxicity (Figure 2A) 176. Moreover, the interaction between HLA-G and LILRB1 disrupted polarization of NK cell lytic granules and the microtubule organizing center (MTOC), along with filamentous actin (F-actin) accumulation at the contact site, severely impaired NK cell cytotoxicity (Figure 2A) 177. Additionally, LILRB1 binding HLA-G expressed on endothelial cells attenuated the rolling adhesion capacity of NK cells (Figure 2A) 178.

### 4.2 Adaptive immunity

Innate immunity serves as the first line of defense, rapidly detecting and eliminating pathogens. In contrast, adaptive immunity employs sophisticated mechanisms to distinguish self from non-self, ensuring precise and specific immune responses 179, 180. This system relies on the orchestrated interplay of APCs, T lymphocytes, and B lymphocytes 180. Crucially, LILRBs serve as key immunoregulators, modulating both T- and B-cell functions through inhibitory signaling.

LILRB1 is broadly expressed on the surface of most T cells and within the cytoplasmic vesicles of all T lymphocytes 181, 182. When LILRB1 was blocked with specific mAbs, the proliferation of antigen-specific polyclonal CD4+ T cells significantly increased, along with enhanced secretion of IL-2, IFN-γ, and IL-13 183. Mechanistically, LILRB1 suppresses T-cell activation by recruiting SHP1/2, which dephosphorylate the TCR-ζ chain, thereby inhibiting the ZAP70-mediated downstream signaling cascade (Figure 2A) 184, 185. In addition, human SEMA4A, expressed on APCs, bound to LILRB2 expressed on CD4+ T cells, enhancing their activation and facilitating Th2 cell differentiation (Figure 2A) 77. Both LILRB1 and LILRB2 can compete with CD8 to bind MHC-I, thereby restricting T-cell activation signals (Figure 2A) 186. While immunogenic DCs drove naïve T-cell differentiation into effector subsets, high LILRB2 expression promoted a tDC phenotype 147. As previously discussed, these tDCs potently suppress T-cell differentiation and function, reinforcing immune tolerance. In summary, LILRBs regulate T-cell differentiation, activation, and function via multiple pathways, maintaining a critical balance between immune tolerance and inflammatory responses.

B lymphocytes play a central role in immunity through antibody production and secretion of immunomodulatory cytokines that regulate both innate and adaptive immune cells 187. Emerging evidence implicates LILRBs in B-cell biology, with elevated expression closely linked to B-cell differentiation 188. While LILRB1 is ubiquitously expressed in peripheral B cells, it indirectly regulates B-cell activity via modulating APC and T-cell responses 20, 188. To date, LILRB1 remains the only family with well-defined functions in B cells 20. Both *in vitro* and *in vivo* studies demonstrated that HLA-G binds to LILRB1 to inhibit B-cell proliferation, differentiation, and Ig secretion (Figure 2A) 189. In addition, elevated sHLA-G in acquired aplastic anemia suppressed BM B-cell proliferation via LILRB1 engagement. Importantly, blocking HLA-G/LILRB1 interactions restores normal proliferative capacity of B cells in BM 190.

### 4.3 Tumor immunity

Cancer represents a complex ecosystem comprising tumor cells and diverse non-tumor elements. These non-tumor cells, embedded within the tumor extracellular matrix, collectively form a dynamic environment that includes immune cells, cancer-associated fibroblasts, endothelial cells, pericytes, and other tissue-specific cell types 191. Dynamic interactions between tumor cells and the TME critically drive tumorigenesis and malignant progression 192. While effective immune responses can eliminate or suppress malignant cells, tumor frequently evade immune surveillance through diverse mechanisms that compromise immune effector functions and subvert anti-tumor immunity 9, 193, 194. LILRBs are predominantly expressed on myeloid-derived cells within the TME, and their signaling activation can promote tumor immune escape 20, 156, 195, 196. Aberrant LILRB expression has also been observed in various cancers, particularly hematological malignancies 22, 47, 116, 156, 195-200. In such contexts, LILRBs may directly regulate cancer initiation, drug resistance, recurrence, and the activity of cancer stem cell 97. Below, we concisely overview LILRB and ligand expression patterns, multifaceted roles, and potential mechanisms in both tumor immune cells and tumor cells.

#### Crosstalk between tumor cell-derived ligands and LILRB-expressing immune cells

Cancer arises from genomic instability, which generates tumor antigens through mutations and structural alterations, thereby eliciting cellular immune responses 9. Effective tumor-specific CD8+ T-cell responses depend on recognition of tumor antigens presented by MHC-I or HLA molecules 201. The interaction between MHC-I and immune cells within the TME exhibits a dual role. While tumors commonly evade CD8+ T-cell surveillance by downregulating classical MHC-I expression, overexpression of β2m-complexed MHC-I heavy chains can engage LILRBs on immune cells—including NK cells and TAMs, releasing a "Don't Eat Me" signal that suppresses innate immune functions (Figure 2B) 37, 38. Although classical HLA I molecule downregulation is a common feature in many cancers, elevated surface HLA-G and increased plasma sHLA-G are observed in both hematological and solid malignancies (Figure 2B) 202, 203. For instance, a proportion of gastric cancers (GCs) exhibited HLA-G/sHLA-G expression 204-206. HLA-G overexpression corelated with decreased number of NK cells through inhibiting the cell proliferation and cytotoxic activity, as well as reducing IFN-γ and TNF-α secretion through its interaction with LILRB1 205. Similarly, elevated sHLA-G in pancreatic cancer inversely correlated with peripheral activated T cells (Figure 2B) 207. Collectively, tumor cells exploit interactions with LILRB-expressing immune cells via β2m, sHLA-G, and membrane-bound HLA-G to induce immune tolerance at various stages of the immune response 50, 202.

#### The dual role of LILRBs in tumor immunity and progression

LILRBs are primarily expressed on myeloid and certain hematopoietic cells, where they critically regulate immune responses 20, 21. However, recent studies revealed their abnormal expression in tumor cells themselves, directly contributing to tumor maintenance and progression 208. Aberrant LILRB expression is well-documented in hematological malignancies, including AML 209-212, chronic monocytic leukemia 213, and MM 214, 215, where genetic knockdown of LILRBs significantly inhibits proliferation and migration. Conversely, lymphocyte-derived tumors, such as chronic lymphocytic leukemia (CLL) 216, acute lymphoblastic leukemia (ALL) 217, and acute T-cell leukemia (ATL) 89 exhibit reduced LILRB expression. In solid tumors, LILRBs are specifically expressed or upregulated across diverse lineages: digestive 218-221, respiratory 222-224, reproductive 219, 225, urinary 226, 227, and nervous system 118, with expression levels correlating closely with disease progression and prognosis. Mechanistically, tumor-expressed LILRBs drive key oncogenic functions, including proliferation, migration, angiogenesis, and metastasis, through activation of multiple pathways such as SHP2/calcium/calmodulin-dependent protein kinase 1 (CaMK1) 72, 223, 228, PI3K/AKT 224, 229, MAPK/ERK 230, 231, NF-kB 230, 232, 233, and JAK/STAT 118, 197 (Figure 2B).

In summary, LILRBs exhibit a complex dual immunoregulatory role in tumor immunity. As inhibitory receptors on immune cells, they engage tumor-expressed ligands, to trigger immunosuppressive signaling that establishes an immunosuppressive milieu. Conversely, aberrant LILRB expression in tumor cells directly drives tumor proliferation, migration, and metastatic dissemination through activation of diverse oncogenic signaling cascades. These findings collectively establish LILRBs as critical determinants of TME dynamics and highlight their significant therapeutic promise in oncology.

## 5. Roles of LILRBs in various diseases

### 5.1 LILRBs and infectious diseases

LILRBs critically regulate immune responses by suppressing effector functions of DCs, macrophages, MDSCs, and NK cells. Pathogens have evolved mechanisms to exploit this immunosuppressive axis to evade the host immunity. Disruption of pathogen-associated ligand-LILRBs interactions can shorten infection cycles and reduces pathogen survival 18. Understanding how LILRB-mediated immune evasion provides valuable insights into the pathogenesis of infectious disease and informs the development of potential immune-based therapeutic strategies. The following sections detail the major mechanisms by which LILRBs modulate infectious disease outcomes (Table 1).

#### Viral infections

##### Cytomegalovirus (CMV)

CMV, a β-herpesvirus, encodes immunomodulatory genes that subvert both innate and adaptive immunity 234. While establishing lifelong latency in immunocompetent hosts, CMV caused severe complications in immunocompromised individuals 235. The viral UL18 protein shares structural homology with MHC-I and exhibits high-affinity binding to LILRB1 (but weak binding to LILRB2) on immune cells 59, 61. During early infection, UL18 inhibited cytotoxicity of LILRB1+ NK cells, thereby protecting infected cells from destruction 109. Notably, UL18 also activated a subset of LILRB1-NK cells through a LILRB1-independent mechanism, which can counterbalance the inhibitory effect of UL18 on LILRB1+ NK cells in the global NK cell response 236. Furthermore, UL18 engaged LILRB1 on DCs, impairing DC maturation and migration, and inhibiting subsequent allogeneic T-cell proliferation 237. Beyond immune evasion, CMV manipulates cell death pathways to facilitate viral spread. Crucially, LILRB1+ cytotoxic T cells lysed UL18+ CMV-infected cells, while cells lacking UL18 were resistant to this effect. This process was independent of antigen specificity and MHC restriction, and it can be blocked by mAbs targeting LILRB1 and UL18 238. Clinically, CMV infection is a common complication following organ transplantation, and LILRB1 upregulation on lymphocytes was associated with CMV reactivation post-lung transplantation 239, suggesting its utility as an early biomarker in transplant recipients.

##### Human immunodeficiency virus type 1 (HIV-1)

HIV-1 infection triggered profound immune dysregulation in its early stages 240. Alterations in LILRB expression critically impaired DC and NK cell function in HIV-1+ individuals 241, 242. During early infection, specific HLA-B variants engaged LILRB2 on monocytes, inducing tolerance that exacerbates DC dysfunction in chronic infection, impairing DC maturation and expression of co-stimulatory molecules 241. The co-expression of LILRB2/MHC-I on classical DCs during HIV/simian immunodeficiency virus (SIV) infection drove cellular dysregulation, blunting adaptive immunity and hindering viral control 243. As the infection progresses, elevated secretion of HLA-G from monocytes/DCs bound to LILRB2 on DCs, suppressing antigen presentation while promoting proinflammatory cytokine release 244. In untreated HIV-1 patients, strong LILRB2-HLA-I binding correlated with increased viral replication and further DC dysfunction 40. Strikingly, elite controllers, a subset of HIV-1-infected individuals who maintained undetectable viral loads without antiretroviral therapy, exhibited upregulated LILRB1 and LILRB3 on circulating myeloid DCs (mDCs) compared to progressive-stage patients 245-247. Concurrently, expanded LILRB1+ NK cells emerged during viremia and treatment interruptions 19, 85, 248—consistent with NK cells' critical role in killing infected cells and cytokine-mediated viral control. These findings elucidate key HIV-1 pathogenesis mechanisms and reveal potential LILRB-targeted strategies for immune intervention.

##### Dengue virus (DENV)

DENV exploited Fc-gamma receptors (FcγR) for cellular entry *via* antibody-dependent enhancement, amplifying viral load 249, 250. Concurrently, DENV engaged LILRB1 to activate SHP1, which suppressed FcγR-mediated Syk pathway. This inhibition downregulated interferon-stimulated genes, enabling DENV early immune evasion and enhanced viral replication 86. Furthermore, LILRB1 impaired lysosomal acidification necessary for enzyme activation following DENV uptake via SHP1-dependent mechanisms, facilitating evasion of cell-autonomous immunity and promoting intracellular survival 251. Clinically, DENV-infected patients exhibited hyporesponsive CD19+CD27- naïve B cells to Toll-like receptor (TLR)/anti-IgM stimulation. This functional impairment correlated with upregulated expression of inhibitory receptors CD32 (FcγRIIb) and LILRB1 on B cells, likely contributing to disrupted humoral immunity 252.

##### Severe acute respiratory syndrome coronavirus 2 (SARS-CoV-2)

SARS-CoV-2, the causative agent of COVID-19, typically manifests with clinical symptoms such as fever, cough, and myalgia 253. Critically, disease severity in patients correlated with upregulated LILRB4 expression 254. MDSCs exacerbated pathological inflammation through the co-expression of programmed cell death ligand 1 (PD-L1), LILRB4, and IDO-1, functioning as significant producers of proinflammatory cytokines IL-6 and IL-10 255. In murine SARS-CoV-2 infection models, rapid disease progression characterized by neurotropic infection and encephalitis coincided with marked LILRB4 upregulation 256.

##### Other viruses

Beyond the established viral models, LILRBs critically regulate immune evasion and viral dissemination in various other viral infections. In Epstein-Barr virus (EBV)-infected individuals, elevated LILRB1 expression on virus-specific CD8+ T cells impaired anti-viral immunity by suppressing IFN-γ production 182, 257. During chronic hepatitis B progression—particularly in immune-tolerant, active hepatitis, and HBeAg-negative phases—CD56^dim^CD16+ NK cells exhibited significant LILRB1 upregulation. This correlated with reduced cytolytic capacity, decreased IFN-γ production, and increased apoptosis 258. Genetic analyses further indicated that LILRB1/HLA-G variants increase the risk for mother-to-fetus Zika virus transmission, while protective LILRB2 polymorphisms reduce in utero transmission likelihood 108. Notably, the murine LILRB2 homolog, PirB facilitated binding and internalization of neurotropic reovirus serotype 3, promoting central nervous system (CNS) replication and pathogenicity 259.

#### Bacterial infections

##### Mycobacterium tuberculosis (M. tuberculosis)

In active pulmonary tuberculosis, CD56^dim^CD16+ NK cells exhibited significantly elevated LILRB1 expression versus latent tuberculosis infection controls. This upregulation mediated functional suppression—evidenced by reduced CD107a degranulation, impaired IFN-γ production, and increased spontaneous apoptosis—directly impairing anti-mycobacterial immunity 260. Therapeutically, monoclonal antibody blockade of LILRB2 reprogramed human MDSCs toward a proinflammatory phenotype, enhancing *M. tuberculosis* killing capacity and nominating LILRB2 as a therapeutic target 261. Notably, LILRB4 upregulation in latent tuberculosis served as both a diagnostic biomarker and reactivation predictor 262. Furthermore, exposure to* M. tuberculosis* induced monocyte LILRB5 expression, which facilitates direct bacterial binding and activates cytotoxic T-cell proliferation through downstream immunomodulatory signaling 263.

##### Staphylococcus aureus

LILRBs play a crucial role in *Staphylococcus aureus* infections. Murine PirB, along with human LILRB1 and LILRB3, function as direct pathogen recognition receptors for *Staphylococcus aureus*. PirB bound to *Staphylococcus aureus* and interacted with TLRs, promoting inhibitory cytokine release, actively suppressing anti-inflammatory responses 264. Furthermore, LILRB3 impaired neutrophil-mediated defense by suppressing effector functions and microbicidal activity, attenuating IgA-induced ROS production, and inhibiting antimicrobial peptide release 169.

##### Other bacterial infections

LILRBs play pivotal roles in modulating immune responses and inflammation during infections with *Salmonella* 131, *Escherichia coli* 265, and *lepros*y 266. Following TLR recognition of *Salmonella* or its components, upregulated LILRB2 and LILRB4 expression on APCs promoted immune tolerance 131, with LILRB4 additionally suppressing T-cell activation and reducing IL-8 production through cell-contact-dependent mechanisms 131. During *Escherichia coli* infections, lipopolysaccharide (LPS) induced macrophage expression of PirB, subsequently downregulating proinflammatory cytokine production 265.

##### Sepsis

Sepsis induces profound immune paralysis, impairing bacterial clearance while triggering dysregulated inflammation. Critically, LILRB2 upregulation on monocytes correlated with immunosuppression and organ failure in patients, characterized by diminished CD86 expression and elevated IL-10/IL-12 ratios 267. Paradoxically, neutrophils failed to upregulate LILRB2, impairing phagocytosis and suggesting LILRB2 modulation could prevent neutrophil dysfunction 167. In septic shock, LPS-induced dysregulation of LILRB2 predicted mortality risk, establishing its central immunomodulatory role 268. Concurrently, heightened LILRB3 expression in peripheral blood mononuclear cells (PBMCs) and macrophages suppressed bacterial killing, ROS production, and antigen presentation, dampening Th1 immune responses 269. Therapeutic blockade of LILRB3 enhanced bacterial clearance, ROS generation, and Th1 differentiation, improving survival in preclinical models 269. Neonatal sepsis further demonstrated significant LILRB2, LILRB3, and LILRB4 upregulation, with LILRB4 driving immune suppression through conversion of effector T cells into suppressive phenotypes 270.

#### Parasite infections

##### *Plasmodium falciparum* (*P. falciparum*)

*P. falciparum* malaria remains a leading global infectious threat, with severe disease manifestations posing significant clinical challenges 271. The parasite evades the host immune system by expressing variant RIFIN proteins on erythrocytes, which interact with immune inhibitory receptors to facilitate escape 272. Specifically, RIFIN mimicked HLA-I structures to bind LILRB1, suppressing NK cell cytotoxicity and inhibiting B-cell IgM production, enabling immune detection avoidance in severe malaria 63, 64. RIFINs additionally bound LILRB2, amplifying evasion through redundant HLA-I mimicry 66. Chronic exposure expanded atypical CD56- NK cells that exhibited elevated LILRB1 expression and enhanced *P. falciparum*-specific antibody-dependent cellular cytotoxicity compared to CD56^dim^ NK cells 273. In malaria patients, increased LILRB1 on CD19+ B cells correlated with early apoptosis markers and excessive cytokine release, potentially impairing immunological memory 274. Additionally, infants born to mothers with placental malaria showed LILRB2 overexpression on non-classical monocytes, highlighting its potential role in mediating malaria-induced immune tolerance 275.

##### *Toxoplasma gondii* (*T. gondii*)

*T. gondii* infection is typically asymptomatic in immunocompetent individuals but can cause adverse pregnancy outcomes in immunocompromised hosts, particularly pregnant women 276. *T. gondii* infection dysregulated KIR2DL4/LILRB1 on decidual NK cells and HLA-G on trophoblasts, inducing excessive immunotolerance that suppressed NK function and facilitated fetal infection, ultimately contributing to adverse pregnancy outcomes 277. Furthermore, *T. gondii* infection downregulated LILRB4 on decidual MDSCs by inhibiting STAT3 phosphorylation and altered arginase-1 and IL-10 expression via the SHP2/STAT6 pathway. These alterations disrupted decidual MDSC immunosuppressive function, suggesting a pivotal role in adverse pregnancy outcomes 278.

##### Trypanosoma cruzi

In chronic *Trypanosoma cruzi* infection, LILRB1 expression was increased during specific CD4+ T cell differentiation. This upregulation impaired T cell function, compromising anti-parasite immune responses and potentially fostering persistent chronic infection 279.

### 5.2 LILRBs and autoimmune diseases

Autoimmune diseases arise from immune dysregulation, triggering activation of autoreactive immune cells and subsequent tissue damage 280. LILRBs critically modulate immune tolerance and effector responses, contributing to autoimmune pathogenesis. Genetic polymorphisms and deletions in LILRBs are correlated with disease susceptibility and altered Treg development 18, 94. Below, we detail the pathogenic roles of LILRBs in major autoimmune disorders (Table 1).

#### Thyroid-related immune diseases

Pathogenic dysregulation of LILRBs is especially pronounced in thyroid diseases. In autoimmune thyroid diseases (AITD), LILRB1 expression was elevated on lymphocytes, but its inhibitory capacity on CD4+ and CD8+ T cell proliferation was impaired. This functional impairment extended to IL-10 regulation, a critical anti-inflammatory cytokine that suppresses cellular immunity. AITD patients demonstrated reduced LILRB1-mediated IL-10 synthesis, potentially disrupting immune homeostasis 281. Notably, the LILRB1 missense variant c.479G>A (p.G160E) was associated with familial autoimmune disorders including Graves' disease, Hashimoto's thyroiditis, and systemic lupus erythematosus (SLE). This variant reduced LILRB1 expression on Treg cells, disrupting SHP1 signaling and promoting pro-inflammatory M1-like macrophage expansion, thus disturbing the M1/M2 balance 282.

#### Rheumatoid arthritis (RA)

In RA patients with CMV co-infection, the interactions between UL18, HLA-G, classical MHC-I molecules, and LILRB1 were disrupted. Declining sHLA-G levels impaired LILRB1-mediated suppression of CD8+ T cells, potentially triggering pathogenic hyperactivation and accelerating RA progression 283. Paradoxically, some research has demonstrated that even when plasma sHLA-G concentrations increase in RA, LILRB1 often fails to recognize it due to HLA-G's preferential formation of monomeric or non-canonical conformations, rather than the dimeric structure required for productive receptor engagement. This structural mismatch prevented LILRB1 from exerting its immunosuppressive functions, perpetuating chronic inflammation 284.

#### Systemic lupus erythematosus (SLE)

In SLE, LILRB1 function was compromised in PBMCs, with significantly reduced surface expression on B lymphocytes—a perturbation implicated in SLE pathogenesis 285. Furthermore, the LILRB4 rs11540761 single nucleotide polymorphism (SNP) associated with downregulated receptor expression on circulating monocytoid DCs, establishing LILRB4 dysregulation as a key mechanism in SLE 106. Elevated LILRB4 expression on plasmablasts and plasma cells in untreated SLE patients suggested that LILRB4 inhibition could suppress pathological autoantibody production, presenting a novel therapeutic strategy 286.

#### Multiple sclerosis (MS)

In healthy CNS tissue, LILRB1 expression was minimal or absent, but in MS lesions, both LILRB1 and its ligand HLA-G were upregulated, modulating neuroinflammatory responses 287. Glatiramer acetate (GA; Copaxone), a copolymer therapeutic agent approved by the US Food and Drug Administration (FDA) for relapsing-remitting MS, engaged murine PirB on MDSCs to regulate myeloid function. Crucially, human LILRB2 and LILRB3 serve as functional orthologs of PirB mediating GA binding. GA competitively occupied LILRB2/LILRB3 in dose-dependent fashion, restoring immune homeostasis 288. Additionally, recombinant human LILRB4 protein has shown promise in MS mouse models by suppressing proinflammatory cytokine release and inhibiting pathogenic Th1 and Th17 cell proliferation, preventing autoimmune neuroinflammation and suggesting a potential treatment modality for MS 289.

### 5.3 LILRBs and neurodegenerative diseases

In AD, soluble Aβ oligomers mediated cognitive dysfunction and synaptic loss 290. Both murine PirB and its human homolog LILRB2 functioned as receptors for Aβ_1-42_ oligomers in CNS tissue (Table 1). This interaction mediated synaptic toxicity and induced early deficits in developmental visual cortex plasticity in AD models, suggesting that blocking LILRB2 could be a promising therapeutic strategy for AD 291. Additionally, microglia contributed to AD progression through LILRB2-TREM2 interactions, which inhibited TREM2 signaling and compromised microglial function 292. Notably, LILRB4 mAbs have been shown to enhance microglial activation and Aβ phagocytosis while suppressing interferon pathways 84, positioning LILRB4 blockade as a complementary immunomodulatory approach for AD.

### 5.4 LILRBs and tumors

LILRBs exert central immunomodulatory functions across diverse malignancies, critically influencing tumor development and progression. This section comprehensively analyzes LILRB expression patterns in human cancers, evaluates their associations with clinicopathological features and survival outcomes, and elucidates their mechanistic contributions to tumorigenesis and metastatic dissemination (Table 2). Collectively, these findings establish LILRBs as master regulators of cancer immunoediting and highlight their potential as therapeutic targets and prognostic biomarkers.

#### Hematological malignancies

##### Acute myeloid leukemia (AML)

**Figure 3:**
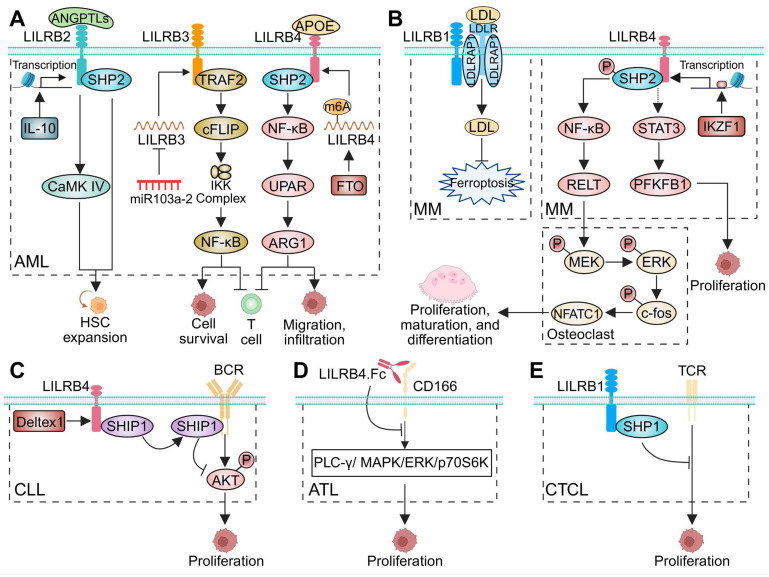
** Roles of LILRBs in hematological malignancies.** (A-E) The schematic diagrams illustrate the functional roles of LILRBs in hematologic malignancies, depicting their interactions with specific ligands, activation of downstream signaling pathways, and subsequent modulation of tumor cell proliferation, migration, and T-cell function across distinct disease contexts: (A) acute myeloid leukemia (AML), (B) multiple myeloma (MM), (C) chronic lymphocytic leukemia (CLL), (D) acute T-cell leukemia (ATL), and (E) cutaneous T-cell lymphoma (CTCL).

LILRBs serve as critical pathogenic regulators in AML, orchestrating tumor progression and immune remodeling within the TME. Multiple studies have demonstrated that LILRB1-4 are highly expressed in AML cells, particularly in monocytic differentiation-associated AML (M-AML) 22, 72, 198, 210-212, 233, 293-295. Among these, LILRB1 and LILRB4 co-expression served as a specific diagnostic marker to differentiate M-AML from other subtypes 209. Elevated LILRB1-4 correlated with poor prognosis and promoted leukemogenesis, while conversely, higher LILRB5 expression was associated with improved outcomes 97, 212. Within the AML TME, NK cells exhibited marked upregulation of LILRB2 and LILRB3 296. Mechanistically, IL-10-driven LILRB2 expression in HSCs facilitated ANGPTL-mediated activation of SHP2 and CaMK pathways, promoting leukemic progenitor expansion and disease progression (Figure 3A) 72, 211, 293. LILRB3 recruited TNF receptor-associated factor 2 (TRAF2) and cellular FLICE-inhibitory protein (cFLIP) to activate NF-κB, supporting AML survival while suppressing T-cell function (Figure 3A) 233. LILRB3 agonism enhanced cholesterol metabolism, supporting AML cell survival 198. Conversely, LILRB3-targeted approaches—including monoclonal antibodies and CAR T cells—have demonstrated potent anti-AML cytotoxicity *in vitro*. Moreover, LILRB3-specific CAR T cells induced durable remission *in vivo*, with transgenic models showing only mild monocytopenia and no significant hematologic or metabolic toxicity 198. Additionally, miR-103a-2-5p has been shown to downregulate LILRB3 and inhibit AML cell growth (Figure 3A) 120. LILRB4 expression was regulated post-transcriptionally by m6A methylation, with FTO promoting its upregulation (Figure 3A) 119. Another study suggested that APOE activates LILRB4 and modulates the SHP2/NF-κB/urokinase plasminogen activator receptor (UPAR)/Arginase-1 (ARG1) axis (Figure 3A), facilitating AML cell migration, infiltration, and T-cell suppression 22. Clinically, the rs1048801 variant in LILRB4 was associated with refractory disease, poorer treatment response, reduced overall survival (OS), and may serve as an independent prognostic risk factor in AML 111.

##### Chronic myelomonocytic leukemia (CMML) and myelodysplastic syndromes (MDS)

LILRB4 mRNA expression was significantly upregulated in CMML compared to MDS and healthy controls. This upregulation was closely associated with activation of pivotal immune checkpoint pathways, including PD-1, cytotoxic T-lymphocyte antigen 4 (CTLA-4), IFN-γ response, and inflammatory signaling, indicating LILRB4-mediated immune dysregulation in CMML pathogenesis 213. While similar associations were observed in MDS, they appeared less pronounced. Additionally, LILRB4 expression positively correlated with CTLA-4 levels, suggesting its involvement in immune evasion via CTLA-4-dependent mechanisms 213.

##### Multiple myeloma (MM)

Patients with active MM and monoclonal gammopathy of undetermined significance (MGUS) exhibited reduced expression of LILRB1 and its ligand S100A9. This impaired LILRB1/S100A9 interaction compromised NK and T-cell cytotoxic activity, contributing to immune evasion and disease progression 214. Abnormal plasma cells from MM patients also exhibited decreased LILRB2 and LILRB3 expression, while LILRB4 and LILRB5 levels remained largely unchanged 214. Contrastingly, a recent study indicated that myeloma cells from MGUS and MM patients exhibit significantly higher LILRB1 levels compared to normal plasma cells from healthy donors 297. Elevated LILRB1 expression was associated with advanced disease stages, high-risk cytogenetic abnormalities (such as t(4;14) translocation), and unfavorable survival outcomes 297. Mechanistically, LILRB1 promoted LDL uptake through interactions with the LDL receptor (LDLR) and LDL receptor adapter protein 1 (LDLRAP1), maintaining metabolic homeostasis and conferring ferroptosis resistance (Figure 3B) 297. Furthermore, LILRB1 was highly expressed on NK cells in MM patients, and its blockade significantly enhanced NK cell cytotoxicity 298.

Concurrently, LILRB4 expression was notably high in MM and linked to worse prognosis 116, 215, 232, 299. Functionally, LILRB4 promoted myeloma cell proliferation and migration, whereas its silencing inhibited disease progression both *in vitro* and *in vivo* 116, 215. Transcription factor IKZF1 activated LILRB4 expression, driving MM cell proliferation via the STAT3/PFKFB1 pathway (Figure 3B) 116. Additionally, high LILRB4 expression correlated with bone destruction severity. Mechanistically, LILRB4 recruited phosphorylated SHP2 to activate NF-κB signaling, promoting multiple myeloma cells to secret RELT. This, in turn, stimulated osteoclast proliferation, maturation, and differentiation via the phosphorylation of MEK/ERK/c-fos and NFATC1 pathways (Figure 3B) 232.

##### Chronic lymphocytic leukemia (CLL)

LILRB1 expression was significantly reduced on leukemia cells in CLL patients, but notably increased on NK cells, particularly in those with advanced disease or adverse prognostic features, such as del(11q) and trisomy 12 216. Additionally, LILRB4, absent in normal B cells, was detected in approximately 50% of CLL patients and was typically associated with advanced lymphoid involvement. In LILRB4-expressing CLL cells, co-expression of LILRB2 was frequently observed, displaying similar expression patterns 300. Mechanistically, LILRB4 expression in CLL was regulated by Deltex1, an inhibitor of antigen receptor signaling. LILRB4 recruited Src homology 2 domain-containing inositol phosphatase 1 (SHIP1), inhibiting B-cell receptor (BCR)-dependent AKT signaling and thereby driving tumor progression (Figure 3C) 301.

##### Acute lymphoblastic leukemia (ALL)

Gene expression profile and experimental validation of CD10+CD19+ precursor B lymphoblasts in pediatric ALL revealed significant LILRB2 downregulation in leukemia cells 302. In contrast, KMT2A-rearranged ALL cells that developed resistance to the DOT1L inhibitor pinometostat exhibited upregulated LILRB4, suggesting myeloid-like phenotypic shifts in resistant populations 217.

##### Acute T-cell leukemia (ATL)

In ATL, LILRB4.Fc inhibited tumor growth by suppressing the PLC-γ/MAPK/ERK/p70S6K signaling pathway (Figure 3D) and disrupting the homophilic CD166-CD166 intercellular adhesion, thereby interfering with the production and acquisition of growth factors 89.

##### Cutaneous T-cell lymphoma (CTCL)

LILRB1 was highly expressed in CTCL subtypes, including CD56+, CD8+, CD56+CD4+, and Sézary syndrome 303, 304. Functional study demonstrated that LILRB1 activation recruits SHP1, which specifically inhibited CTCL cell proliferation induced by CD3/TCR stimulation (Figure 3E) 305.

##### Other lymphomas

LILRB expression has been confirmed in multiple lymphoma subtypes 306-308. Bioinformatics analyses revealed significantly elevated LILRB2 expression in classical Hodgkin lymphoma, correlating with poor prognosis 309. Furthermore, M2-like TAMs within the TME of follicular lymphoma and diffuse large B-cell lymphoma harboring BCL6 translocations exhibited high LILRB3 expression 310. In elderly EBV-positive diffuse large B-cell lymphoma patients, a higher frequency of LILRB1 mutations was observed 311.

#### Digestive system

##### Esophageal cancer

**Figure 4:**
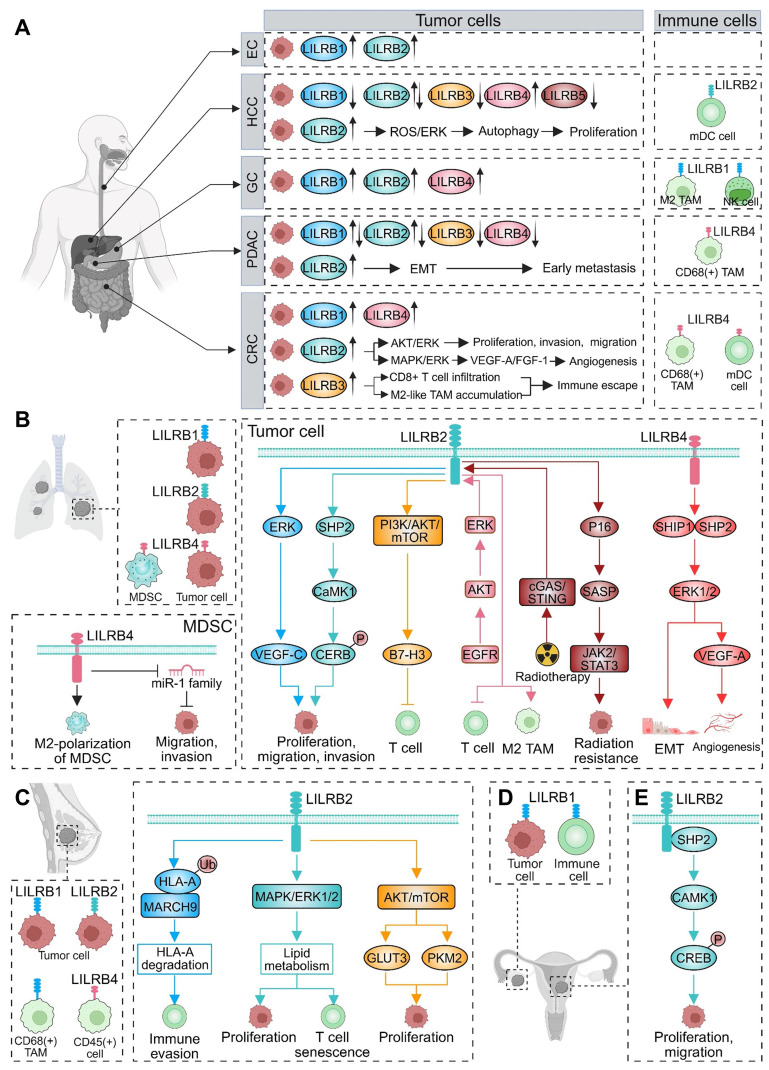
** Roles of LILRBs in digestive, respiratory and reproductive cancers.** (A) Expression patterns and functional roles of LILRBs in esophageal carcinoma (EC), hepatocellular carcinoma (HCC), gastric cancer (GC), pancreatic ductal adenocarcinoma (PDAC), and colorectal cancer (CRC). The diagrams illustrate their distribution and functions in tumor cells (left) and immune cells (right). In HCC and CRC, LILRB2 regulates proliferation, migration, invasion, epithelial-mesenchymal transition (EMT), and angiogenesis through the ROS/ERK, AKT/ERK, and MAPK/ERK signaling pathways. In CRC, LILRB3 promotes immune escape by modulating the spatial distribution of CD8+ T cells and M2-type tumor-associated macrophages (TAMs) within the tumor microenvironment. (B) In NSCLC, LILRB2 regulates tumor proliferation, migration, invasion, and influences the immune microenvironment through the ERK, SHP2-CaMK1, JAK-STAT3, and PI3K-AKT signaling pathways, thereby promoting tumor progression. Additionally, LILRB2 expression is regulated by EGFR and cGAS-STING pathways. LILRB4 promotes EMT and angiogenesis in NSCLC by activating ERK pathway. In MDSCs, LILRB4 enhances MDSC-mediated tumor metastasis by regulating M2-type macrophage polarization and suppressing the secretion of miR-1 family miRNAs. (C-E) LILRB expression patterns in (C) breast cancer, (D) ovarian cancer, and (E) endometrial cancer, along with their key downstream signaling pathways.

LILRB1 expression was significantly upregulated in esophageal adenocarcinoma (Figure 4A) 221. Additionally, LILRB2 was notably elevated in esophageal squamous cell carcinoma and early-stage esophageal adenocarcinoma (pT1-2) (Figure 4A) 312.

##### Hepatocellular carcinoma (HCC)

Bioinformatics analyses revealed significant downregulation of LILRB1, LILRB2, LILRB3, and LILRB5 mRNA in HCC tissues, whereas LILRB4 was notably upregulated (Figure 4A) 313. Additionally, the expression of LILRB family members correlated with patient prognosis and immune cell infiltration 313. Contradicting this, another study reported LILRB2 overexpression in HCC tissues, which was associated with poor differentiation, larger tumor size, and reduced OS 314. Subsequent investigations demonstrated that LILRB2/PirB interacts with ANGPTL8 to activate the ROS/ERK pathway, thereby enhancing autophagy and promoting HCC proliferation (Figure 4A) 196. Moreover, LILRB2 expression was significantly elevated in the CD1c+ mDC subpopulation within tumor tissues, particularly in poorly differentiated tumors, suggesting its potential role in suppressing immune responses 315, 316.

##### Gastric cancer (GC)

LILRB1 and LILRB4 were highly expressed in poorly differentiated and undifferentiated GC cells, with LILRB1 levels showing a clear correlation with tumor size and degree of differentiation (Figure 4A) 220. In gastric mixed adenocarcinoma, LILRB1 expression associated with advanced pathological stage and reduced OS 219. LILRB2 was significantly upregulated in GC cells and linked to neutrophil extracellular trap-related transcriptional reprogramming (Figure 4A) 317. Within the TME, LILRB1 was predominantly localized to M2-type TAMs, with high expression predicting advanced tumor stage, increased recurrence risk, and unfavorable prognosis 318. Additionally, HLA-G interacted with LILRB1 to inhibit the proliferation and cytotoxic activity of NK cells, thereby facilitating immune escape in GC 205.

##### Pancreatic ductal adenocarcinoma (PDAC)

In PDAC, LILRB1 expression was significantly elevated in tumor tissue versus adjacent normal tissue, correlating with advanced pathological stage and poor OS 219. During multistep tumorigenesis in PDAC models, LILRB2 and its ligand ANGPTL2 were progressively upregulated. Subsequent studies showed that the ANGPTL2/LILRB2 autocrine axis promotes epithelial-to-mesenchymal transition (EMT) and early metastasis (Figure 4A) 319. Soluble LILRB4, mainly derived from CD68+ TAMs, induced inhibitory differentiation of CD8+ T cells, thereby suppressing antitumor immunity 320. Interestingly, another study found reduced expression of LILRB1-4 in early-stage PDAC tumors compared to adjacent normal tissues, with high LILRB4 levels correlating with improved relapse-free survival (RFS) 321.

##### Colorectal cancer (CRC)

LILRB1+ cells were significantly more abundant in colorectal mucinous adenocarcinoma tumors and inversely correlated with OS 219. Elevated LILRB2 expression was associated with male, poor differentiation, vascular invasion, lymph node (LN) metastasis, and advanced clinical stage 218, 231, 322, 323, and served as an independent prognostic factor for poorer outcomes 231, 323. Mechanistically, LILRB2 promoted CRC progression by interacting with HLA-G, activating AKT and ERK signaling (Figure 4A) 322, enhanced tumor angiogenesis via MAPK/ERK-mediated upregulation of vascular endothelial growth factor (VEGF)-A and fibroblast growth factor 1 (FGF-1, Figure 4A) 231. LILRB3 expression was significantly higher in CRC versus normal colorectal epithelium and associated with LN metastasis, advanced tumor stages and reduced survival 324. Tumor-derived LILRB3 inhibited CD8+ T cell infiltration and polarized TAMs toward an M2-like phenotype, mediating immune escape (Figure 4A) 324. Similarly, high LILRB4 expression correlated with LN metastasis, advanced disease, reduced CD45RO+ T cell infiltration, poor OS, and was an independent prognostic factor 325. In the TME, LILRB4 was prominently expressed on mDCs and CD68+ TAMs, implicating its role in immune suppression (Figure 4A) 320, 326.

#### Respiratory system

##### Non-small cell lung cancer (NSCLC)

LILRB1 expression was detected in over half of NSCLC samples, with levels escalating alongside advancing tumor stage 107. The LILRB1 gene SNP c.5724G>A (p.E625K) was more commonly found in NSCLC patients and significantly associated with regional LN metastasis, although it does not appear to influence LILRB1 expression 107. Compared to adjacent normal tissue, LILRB2 expression was significantly elevated in NSCLC tumor tissue 222-224, 327, and associated with adverse clinicopathological features including poor differentiation, regional LN metastasis, advanced tumor stage, older age, reduced tumor-infiltrating lymphocytes (TILs), and poorer prognosis 222-224, 327-329. Functionally, LILRB2 activated ERK signaling pathway, increasing VEGF-C expression and promoting tumor cell malignancy, migration, and invasion (Figure 4B) 222, 223. Additionally, LILRB2 interacted with ANGPTL2 to activate the SHP2/CaMK1/CREB signaling pathway, further supporting tumor cell proliferation (Figure 4B) 223. In terms of immune evasion, LILRB2 promoted B7-H3 expression via PI3K/AKT/mTOR signaling to suppress T cell-mediated anti-tumor immunity, aiding NSCLC progression (Figure 4B) 224. LILRB2 expression was further regulated by EGFR/AKT/ERK pathways, facilitating recruitment of M2-type TAMs and inhibiting T cell activity (Figure 4B) 199. Notably, radiotherapy increased LILRB2 expression through cGAS/STING pathway, promoting cellular senescence and activating the JAK2/STAT3 axis, which drives tumor progression and radiation resistance (Figure 4B) 197. LILRB4 was also highly expressed in NSCLC and strongly associated with adverse clinical outcomes 230. LILRB4 recruited SHP2 and SHIP1 to activate ERK1/2 pathway, thereby promoting EMT and upregulating VEGF-A expression, which in turn enhances tumor cell migration, invasion, and angiogenesis (Figure 4B) 230. Within the TME, high LILRB4 expression on tumor-infiltrating immune cells predicted increased risk of postoperative recurrence, shorter OS, and reduced RFS, establishing it as an independent prognostic factor 330. LILRB4 was predominantly expressed on MDSCs and its blockade significantly inhibited NSCLC cell migration in co-culture systems with CD33+ MDSCs 330, 331. Furthermore, LILRB4/gp49b regulated MDSCs M2 polarization and suppressed miR-1 family miRNAs secretion, thereby promoting MDSC-mediated metastasis and enhancing tumor migration and invasion (Figure 4B) 332.

#### Reproductive system

##### Breast cancer (BC)

In invasive ductal carcinoma of the breast, LILRB1 expression was significantly higher in tumor cells than in adjacent normal tissue, positively correlating with tumor stage and inversely correlating with OS 219. In the TME of BC, LILRB1 was predominantly expressed by infiltrating CD68+ macrophages 333, and was also notably upregulated in NK cells, particularly in patients with invasive BC relative to healthy donors and individuals with noninvasive (in situ) lesions 334, 335. LILRB2 expression was detected in BC cell lines, as well as in primary ductal and lobular carcinomas, but was absent in normal breast tissue. Its upregulation was associated with reduced TILs, increased LN metastasis 336, and poor prognosis 195, 229. Mechanistically, LILRB2 promoted immune evasion by facilitating the interaction between HLA-A and the E3 ubiquitin ligase membrane-associated RING-CH finger protein 9 (MARCH9), leading to HLA-A degradation (Figure 4C) 195. Furthermore, LILRB2 activated MAPK/ERK1/2 signaling, which increased fatty acid synthesis and lipid accumulation to support tumor progression while inducing T cell senescence (Figure 4C) 229. In triple-negative BC, elevated LILRB2 activated AKT/mTOR signaling to upregulate glucose transporter 3 (GLUT3) and pyruvate kinase muscle 2 (PKM2), thereby reprogramming aerobic glycolysis and enhancing tumor aggressiveness (Figure 4C) 337. LILRB4 expression was notably enriched in infiltrating CD45+ cells in BC relative to other cancers 338. By regulating cytokines like IL-6, IL-1β, and TNF, LILRB4 influenced BC incidence, tumor growth, and patient survival outcomes 339.

##### Ovarian cancer

In ovarian cancer, LILRB1 was highly expressed in both tumor cells and infiltrating immune cells (Figure 4D). A high density of LILRB1+ immune cells correlated significantly with younger age, advanced FIGO stage, shorter survival, and chemoresistance 225. Additionally, LILRB1 expression was linked to increased infiltration of M2-type macrophages, reduced DC activation, and impaired CD8+ T cell function. Notably, the combined assessment of LILRB1+ immune cells and CD8+ T cell levels effectively stratified patient prognosis 225.

##### Endometrial cancer and cervical cancer

LILRB2 was significantly upregulated in endometrial cancer, where it predicted poor prognosis and functionally promoted tumor cell proliferation, colony formation, and migration. Mechanistically, LILRB2 promoted tumor progression by activating the SHP2/CaMK1/CREB axis (Figure 4E) 228. In cervical cancer, transcriptomic profiling identified LILRB2 as a robust diagnostic biomarker capable of distinguishing patients from healthy individuals 340.

#### Urinary system

##### Clear cell renal cell carcinoma (ccRCC)

In a preliminary cohort of ten ccRCC patients, LILRB1 and LILRB2 were each expressed in tumor cells from six cases, with markedly elevated LILRB1 levels in three 227. Both receptors were abundantly expressed within the ccRCC TME. Tumor-infiltrating CD8+ T cells expressed LILRB1, with their function inhibited by HLA-G 341. Additionally, infiltrating LILRB1+CD4+ T cells exhibited high cytolytic properties, but were selectively inhibited by HLA-G+ targets 342. Elevated LILRB2 in peritumoral macrophages enhanced infiltration and stimulated VEGF-C production, driving angiogenesis 343. Bioinformatics analyses revealed LILRB3 overexpression in ccRCC, correlating with poor prognosis and increased immune infiltration 344.

##### Bladder cancer

Soluble LILRB1 and LILRB2 levels were low in healthy individuals but significantly elevated in patients with non-muscle invasive bladder cancer (NMIBC) 345. Additionally, patients with recurrent NMIBC exhibited higher frequencies of circulating LILRB1+CD8+ T cells compared to non-recurrent cases, with this increase emerging as an independent prognostic marker for disease recurrence 226.

##### Prostate cancer (PC)

In PC, LILRB1 expression was elevated in NK cell-infiltrated regions of metastatic patients compared to localized disease and healthy controls. Critically, tumor cells induced LILRB1 expression in NK cells while downregulating activating receptors, including NKp46, NKG2D, and CD16, thereby impairing tumor recognition 346. Additionally, elevated mRNA levels of LILRB2, LILRB3, and LILRB5 were associated with shorter RFS 347.

#### Nervous system

##### Glioma

In glioma, high LILRB1 expression correlated with increased tumor volume and independently predicted poor prognosis, confirming its role as a pathogenic driver. Functionally, LILRB1 promoted tumor cell proliferation, migration, and invasion 118. In a bioinformatics study, increased LILRB1 expression was positively associated with hypomethylation, M2 macrophage infiltration, upregulation of immune checkpoints, and activation of the JAK/STAT signaling pathway 118.

##### Glioblastoma

Glioblastoma, a World Health Organization grade IV malignant glioma characterized by poorly differentiated and highly proliferative pleomorphic astrocytes 348, demonstrated significant upregulation of LILRB1-4 expression in tumor tissues while LILRB5 remains unaltered 349. Among these, LILRB2 expression positively correlated with tumor grade, with high expression independently predicting shorter disease-free survival and OS 350. Within the glioblastoma TME, LILRB1 was predominantly expressed on NK cells, where co-culture with tumor cells induced its expression and impaired NK cell cytotoxicity 349. Concurrently, LILRB2/PirB promoted MDSC formation and expansion through small extracellular vesicle secretion, establishing an immunosuppressive microenvironment 350. In addition, LILRB3 was highly expressed in a subset of monocyte-derived TAMs, with its expression closely associated with the mesenchymal-like cellular state and significantly correlated with poor prognosis 163. Mechanistically, LILRB3 bound to APOE and activated the SHP1 signaling pathway, contributing to the formation of an immunosuppressive TME 163.

#### Cutaneous tumors

##### Melanoma

In melanoma, LILRBs are primarily expressed in immune cells within the TME, with individual members fulfilling distinct roles. LILRB2 showed marked overexpression in melanoma cells compared to normal epithelium 229. Its murine ortholog, PirB, drove tumor proliferation, adhesion, and migration through MAPK/ERK1/2-mediated fatty acid synthesis and lipid accumulation, concurrently driving tumor progression while inducing T cell exhaustion 229. LILRB3 was functionally expressed on MDSCs in patients with melanoma, with its activity ligand-dependently activated by galectin-4 and galectin-7 47. Notably, therapeutic blockade of LILRB3 successfully inhibited immunosuppressive activity of MDSC from melanoma patients 47. LILRB4 predominantly localized to CD68+ TAMs in melanoma, facilitating immune escape by inducing CD8+ T suppressor cells and blunting T cell responses 320. Additionally, LILRB4 demonstrated upregulation on CD45+ immune infiltrates, with expression levels increasing progressively during tumor advancement 338.

##### Merkel cell carcinoma (MCC)

MCC, a rare and aggressive skin cancer typically arising in sun-exposed areas, presents a complex TME enriched with diverse immune infiltrates. Strikingly, all TAM and DC subsets in MCC exhibited significantly elevated expression of LILRB1, LILRB2, and LILRB4 351.

#### Tumors of other systems

##### Oral squamous cell carcinoma (OSCC)

In OSCC, LILRB1 mRNA expression was significantly downregulated compared to control tissues 352. This reduced expression inversely correlated with oxidative stress markers, suggesting that oxidative stress may suppress LILRB1 to promote tumor immune evasion 352.

##### Head and neck squamous cell carcinoma

Cancer stem cells in head and neck squamous cell carcinoma are key drivers of multidrug resistance, metastasis, recurrence, and immunosuppression, with LILRB2 expression notably upregulated in these cells 353. However, the functional role and underlying mechanisms of LILRB2 in cancer stem cells require further investigation.

##### Thyroid carcinoma

In papillary thyroid carcinoma, LILRB1 expression was significantly elevated in tumor tissues relative to matched non-tumor tissues. Its expression positively correlated with pathological stage and inversely with OS 219. In anaplastic thyroid carcinoma, LILRB2 identified as a critical immune checkpoint, with high expression in TAMs which drives immune evasion 354.

## 6. Clinical Trials of Targeting LILRBs in Cancer Therapy

LILRBs serve dual roles as immune checkpoint regulators and tumor-promoting factors, highlighting them as compelling therapeutic targets in oncology. Although LILRB-targeted therapies remain in early stages of development, accumulating preclinical evidence provides a robust theoretical foundation for clinical translation. Currently, mAbs and engineered T cell therapies targeting LILRBs are under clinical investigation, with active clinical evaluation primarily focus on hematologic malignancies and advanced solid tumors. This section summarizes ongoing clinical trials, detailing therapeutic modalities, mechanisms of action, and stages of development (Table 3).

### 6.1 Hematological malignancies

#### LILRB4

**Figure 5:**
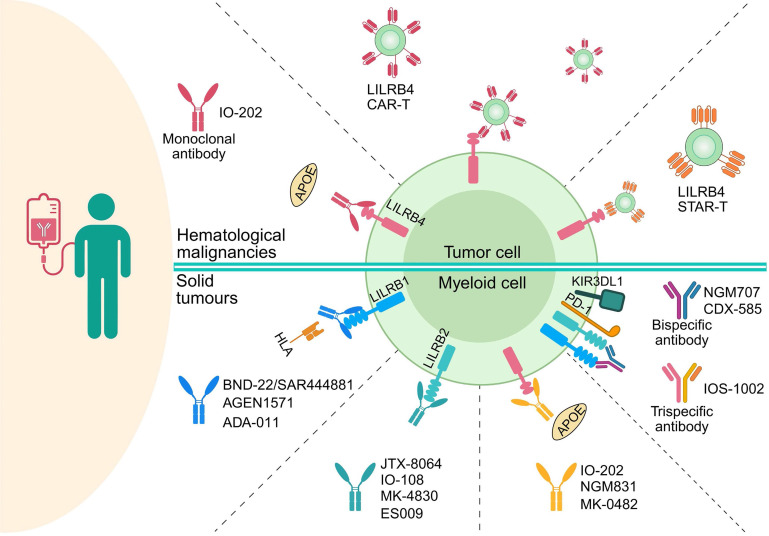
** Clinical trials of LILRB-targeted cancer therapies.** In hematological malignancies, LILRB4-targeted therapeutic strategies include monoclonal antibodies, chimeric antigen receptor (CAR) T-cell therapy, and synthetic T-cell receptor and antigen receptor (STAR-T) therapy, all of which demonstrate potent tumor cell-killing activity. In solid tumors, current approaches employ monoclonal antibodies targeting LILRB1, LILRB2, and LILRB4 to inhibit tumor progression. Additionally, emerging bispecific and trispecific antibodies—designed to simultaneously engage multiple LILRBs or other immune-related proteins—are under development for solid tumors, aiming to enhance antitumor efficacy through synergistic mechanisms.

LILRB4 has emerged as a promising therapeutic target in hematologic malignancies, particularly AML, driving active development of targeted agents. IO-202, a humanized IgG1 mAb, specifically binds LILRB4 and antagonizes its interaction with ApoE, effectively antagonizing downstream signaling and functional effects (Figure 5) 355, 356. This agent has completed evaluation by a Phase I clinical trial (NCT04372433) as a monotherapy or combined with pembrolizumab in AML and CMML patients 355, 356. CAR T-cell therapy—which utilizes genetically engineered T cells expressing CARs to recognize and eliminate tumor cells expressing surface antigens 357. Although six CAR T products are FDA-approved for hematologic malignancies, their development for myeloid malignancies remains limited by the challenge of identifying safe and effective myeloid-restricted targets. LILRB4 represents a compelling CAR T target in AML, due to its selective expression on both leukemia cells and HSCs. A 2018 study demonstrated potent anti-leukemic activity of LILRB4-directed CAR T cells *in vitro* and *in vivo*, with no observed toxicity to normal HSCs (Figure 5) 295. Based on these findings, a Phase I trial (NCT04803929) is currently evaluating this strategy in relapsed or refractory M4/M5 AML subtypes. Recent advances include LILRB4-specific nanoantibodies engineered for Synthetic T-cell Receptor and Antigen Receptor (STAR)-T cells (Figure 5). Preclinically, LILRB4 STAR-T cells effectively eliminated LILRB4+ AML cells 358, with dual-epitope targeting demonstrating enhanced anti-leukemia activity *in vivo*. Following completion of two Phase I STAR-T trials (NCT05518357 and NCT05548088), an additional trial (NCT05739409) is assessing this approach in AML and CMML.

### 6.2 Solid tumors

Development of LILRB-targeted therapies for advanced solid tumors has predominantly focused on LILRB1, LILRB2, and LILRB4. Therapeutic agents against these receptors are progressing through clinical development, with several now in active clinical trials. Current research prioritizes evaluating these therapies as monotherapies, while parallel efforts explore their potential in combination with ICIs, such as anti-PD-1 antibodies.

#### LILRB1

LILRB1 inhibits immune cell activity and amplifies myeloid-driven immunosuppression via interactions with HLA-G. To disrupt this axis, the anti-LILRB1 antibody BND-22/SAR444881 has been developed to block LILRB1 binding to these ligands, thereby restoring immune cell activity (Figure 5) 359. BND-22/SAR444881 is under evaluation in a Phase I/II trial (NCT04717375) to determine its safety, tolerability, and antitumor efficacy, both as a monotherapy and in combination with the anti-EGFR antibody cetuximab or the anti-PD-1 antibody pembrolizumab in patients with advanced solid tumors 359. Several other novel immunotherapies, including combinations with anti-CTLA-4 or anti-PD-1 antibodies, are under clinical evaluation for advanced solid tumors 360. AGEN1571 has completed clinical evaluation in trial NCT05377528, which investigated its efficacy both as monotherapy and in combination with PD-1 inhibitor balstilimab and/or anti-CTLA-4 antibody botensilimab for advanced solid tumors (Figure 5). ADA-011, the third candidate, was evaluated in a Phase I trial (NCT05601219) with/without PD-L1 inhibition before trial termination (Figure 5). ADA-011, the third candidate, was evaluated in a Phase I trial (NCT05601219) with/without PD-L1 inhibition before trial termination. These clinical programs underscore LILRB1 as a therapeutic target and highlight strategic combinations to overcome immune suppression in solid tumors.

#### LILRB2

JTX-8064, a mAb targeting LILRB2, disrupts its interaction with ligands and reprograms macrophages and DCs toward immunostimulatory phenotypes (Figure 5), enhancing anti-PD-1 efficacy in preclinical models 361, 362. Its Phase I trial (NCT04669899) evaluating monotherapy and PD-1 combinations remains unpublished. Similarly, IO-108 induces myeloid proinflammatory reprogramming and synergizes with PD-1 blockade (Figure 5), with Phase I safety/efficacy data (NCT05054348) pending 363. MK-4830, a third LILRB2-targeting mAb, blocks ligand-induced immunosuppression (Figure 5). Early trial data (NCT03564691 and NCT05446870) showed favorable tolerability and dose-dependent target engagement in advanced solid tumors 364. Finally, the high-affinity antibody ES009 reprograms myeloid cells to enhance T-cell activation and potentiates PD-1 inhibitors (Figure 5), has completed Phase I evaluation (NCT06007482) 365. Collectively, these clinical programs validate LILRB2 targeting to reverse myeloid-mediated immunosuppression through phenotypic reprogramming and rational ICI combinations.

#### LILRB4

IO-202 has completed clinical evaluation for AML and CMML, with a separate Phase I trial (NCT05309187) demonstrating its efficacy both as monotherapy and in combination with pembrolizumab for advanced solid tumors (Figure 5). NGM831, designed to disrupt LILRB4 interactions with fibronectin and APOE, attenuating immunosuppression and potentiating myeloid cell activation (Figure 5). It is currently being assessed in a Phase I trial (NCT05215574) for solid tumors. Additionally, MK-0482, an IgG4 mAb targeting LILRB4 has been evaluated across multiple completed trials (NCT03918278 and NCT04165096) to assess safety, pharmacokinetics, and therapeutic efficacy in advanced and metastatic solid tumors (Figure 5). These efforts highlight the therapeutic potential of LILRB4 targeting to reverse tumor immunosuppression and enhance antitumor immunity.

#### Antibodies targeting multiple LILRBs

Bispecific and multi-specific antibodies are advancing clinically for solid tumors, frequently combined with ICIs 366. NGM707, a humanized IgG antibody with dual specificity for LILRB1 and LILRB2, reprograms MDSCs to enhance immune activity (Figure 5). When combined with anti-PD-1 therapy, it further amplifies macrophage-mediated CD4+ T cell activation. NGM707, administered as monotherapy or in combination with pembrolizumab (NCT04913337), is being actively investigated in advanced or metastatic solid tumors. Although the trial is no longer enrolling participants, its results are highly anticipated. CDX-585, a tetravalent bispecific antibody targeting both PD-1 and LILRB2, drives M1 polarization and induces proinflammatory cytokine secretion following treatment with LPS or CD40 agonist mAbs (Figure 5). The Phase I trial of CDX‑585 (NCT05788484) in advanced solid tumors was recently completed, and its forthcoming results are highly anticipated for insights into its clinical efficacy and safety 367. IOS-1002, a trispecific mAb targeting LILRB1, LILRB2, and KIR3DL1, blocks HLA-G/ANGPTL engagement to inhibit ITIM signaling and polarize macrophages toward immunostimulatory phenotype, enhancing tumor phagocytosis (Figure 5) 368. A Phase Ia/Ib clinical trial (NCT05763004) is currently evaluating IOS-1002 both as a monotherapy and in combination with anti-PD-1 antibodies.

Early-phase clinical trials demonstrate promising antitumor activity of LILRB-targeted therapies through remodeling TME, yet their development faces significant challenges. The broad expression of LILRBs across immune and non-immune tissues raises legitimate concerns regarding off-target toxicity and autoimmune sequelae. Compounding this risk, the dual immunomodulatory roles of LILRBs mean that therapeutic interference could disrupt immune homeostasis, leading to unintended immune dysregulation. To advance this therapeutic class, future research must prioritize four critical domains: 1) comprehensive mapping of ligand-receptor interaction networks, 2) single-cell resolution expression profiling across physiological and pathological contexts, 3) mechanism-guided biomarker development for patient stratification, and 4) engineering solutions including optimized Fc domains and conditional activation systems to enhance target specificity. Rigorous safety monitoring integrated with mechanistic pharmacodynamic studies will be essential to maximize therapeutic efficacy while mitigating risks, ultimately enabling transformative cancer immunotherapies.

## 7. Conclusions and Perspectives

This review delineates the pivotal immunoregulatory functions of LILRB family members and their multifaceted contributions to disease pathogenesis. We systematically dissect mechanistic insights into LILRB-mediated TME reprogramming and tumor cell functional modulation across malignancies, while synthesizing clinical advances in LILRB-targeted therapeutic strategies. The compelling capacity of LILRBs to architect immunosuppressive TME and subvert antitumor immunity underscores their emerging potential as therapeutic targets. Future interrogation of LILRB signaling networks promises to unveil novel diagnostic biomarkers, prognostic indicators, and precision immunotherapy paradigms.

Despite advances in understanding LILRB in infectious diseases and tumor, knowledge of their ligands remains limited, hindering a comprehensive analysis of their immune-regulatory mechanisms 18. Current ligand identification methods, including expression cloning, chemical cross-linking with mass spectrometry, high-throughput protein arrays, candidate molecule validation, cell microarrays, and light-based receptor capture techniques, face significant challenges 97, 369, 370. Ligand discovery is complicated by context-dependent expression patterns within specific tumor or disease microenvironments. Moreover, LILRB polymorphisms may alter ligand-binding specificities and contribute to diverse pathological roles. For example, a specific LILRB3 haplotype has been linked to kidney transplant failure and early onset of end-stage renal disease 371, highlighting the broader clinical significance of LILRB genetic variations. These gaps hinder a full mechanistic understanding of LILRB functions and immune regulation 18. Although several downstream signaling pathways activated by LILRB-ligand interactions have been identified 18-20, 24, 372, the overall signaling network remains incompletely defined, particularly in cancer. Future studies elucidating LILRB ligands and their integrated signaling networks will be crucial for unraveling immune-regulatory mechanisms and optimizing cancer treatment strategies.

LILRB expression varies significantly across tumor types and correlates strongly with disease progression 197, 224, 229, 231, 301, 322, 350. However, the dynamic spatiotemporal regulation of LILRBs throughout tumor evolution—from initiation to metastasis—and how different LILRBs cooperate in driving malignancy remain poorly understood 214. Additionally, while LILRBs engage in multiple signaling pathways with extensive crosstalk, the integrated mechanisms through which these networks collectively regulate tumor cell behaviors—including proliferation, apoptosis, invasion, and metastasis—require comprehensive elucidation 72, 116, 215, 222, 223, 228, 232, 305, 321. Furthermore, the mechanisms underlying aberrant LILRB expression in malignancies remain undefined, representing a fundamental barrier to therapeutic development.

LILRBs are widely expressed in immune cells within both hematological and solid tumors 20, 372, where they orchestrate highly complex immunomodulatory functions. Critical knowledge gaps persist regarding how LILRB-mediated intercellular signaling coordinates immune cell crosstalk to balance immune surveillance and tumor evasion 195, 205, 213, 214, 320, 324, 352, 354. The crosstalk between LILRBs and other cytokines or signaling pathways in the TME further complicates understanding their role in immune escape and therapy resistance 296, 310, 320, 324, 326, 333, 342.

Methodological limitations remain a major barrier to advancing LILRB research. The lack of suitable animal models severely restricts the ability to investigate the dynamic regulation and functional roles of LILRBs during disease progression. This challenge is further exacerbated by the limited availability of high-quality research reagents—particularly for understudied members such as LILRB5 49, 313, 373, 374—which significantly hampers mechanistic and functional studies 20, 21. Overcoming these technical deficiencies is essential for deepening our understanding of LILRB biology and enabling its therapeutic exploitation.

To overcome these challenges, future research could prioritize four interconnected areas: First, improving ligand discovery with enhanced detection platforms and integrated methodologies. Second, leveraging single-cell and spatial transcriptomics to map stage-specific LILRB expression trajectories throughout tumor evolution. Third, applying multiplexed proteomic and phosphoproteomic approaches to delineate downstream signaling pathways. Finally, developing 3D organotypic cultures and precision gene-edited *in vivo* models to dissect intercellular LILRB signaling networks. Collectively, these efforts will provide a comprehensive framework for understanding LILRB biology and guiding therapeutic innovation.

Therapeutically, despite the complexity and biomarker challenges, LILRBs remain promising immuno-oncology targets. Rational drug design—such as bispecific antibodies or allosteric inhibitors targeting key LILRB-ligand interactions—may disrupt tumor-immune crosstalk. Clinically, combining LILRB-targeted agents with immune checkpoint blockade or conventional therapies holds potential to overcome resistance and expand durable response rates. As research advances, LILRB-targeted strategies are poised to make transformative impacts in cancer treatment, offering new hope for patients.

## Figures and Tables

**Table 1:** The role of LILRB family in infectious, autoimmune and neurodegenerative diseases.

Diseases	LILRBs	Ligands	Expressing cells	Mechanisms	References
Viral infections					
CMV	LILRB1	UL18	Cytotoxic T cell	Lysis of CMV-infected cells	238
		UL18	NK cell	Inhibit NK cytotoxic activity	236
		UL18	DC	Inhibit DC migration, maturation, and allogeneic T cell proliferation	145, 237
			Lymphocyte	CMV reactivation after lung transplantation	239
HIV-1	LILRB2		Monocyte	Impair monocyte antigen presentation	124
		HLA-B	Myelomonocytic cell	Induce myelomonocytic tolerogenesis	241
		HLA-I	DC	Dysregulation of cDCs	243
		HLA-G	Myeloid DC	Modulate DC antigen presentation and proinflammatory cytokine secretion	244
	LILRB1, LILRB3		Myeloid DC	NA	247
	LILRB1	S100A9	NK cell	Enhance NK cell anti-HIV-1 activity	85
DENV	LILRB1			Enhance DENV replication	86
				Overcome cell-autonomous immunity, boosting DENV intracellular survival	251
				Resistant to TLR/anti-IgM stimulation in CD19+CD27+ naïve B cells	252
SARS-CoV-2	LILRB4		MDSC	Elevated inflammation and regulatory B/T cell accumulation	255
EBV	LILRB1		CD8+ T cell	Inhibiting IFN-γ production to impair immunity	182, 257
CHB virus	LILRB1		NK cell	Reduce NK cell degranulation and IFN-γ production	258
Zika virus	LILRB1, LILRB2			Mutations impact in utero transmission chances	108
Reovirus	LILRB2/PirB			Essential for reovirus replication and neuropathogenicity	259
Bacterial infections					
*Mycobacterium tuberculosis*	LILRB1		NK cell	Decrease CD107a and IFN-γ expression	260
	LILRB2		MDSC	Transition between immunosuppressive and proinflammatory states	261
	LILRB5		Cytotoxic T cell	Enhance cytotoxic T cell proliferation	263
*Staphylococcus aureus*	LILRB1, LILRB3/PirB		Macrophage	Influence TLR-mediated cytokine production	264
	LILRB3		Neutrophil	Regulate neutrophil activation and antimicrobial functions	169
*Salmonella*	LILRB2, LILRB4		Antigen presenting cell	Induce tolerogenic antigen-presenting cells	131
*Escherichia coli*	LILRB1, LILRB4, PirB		Macrophage	Enhanced proinflammatory cytokine production and MAPK/NF-kB activation	265
Parasite infections					
*Plasmodium falciparum*	LILRB1	RIFIN	NK and B cells	Regulate IgM production and NK-mediated killing	63, 64
			B cell	Modulates inflammatory cytokine release	274
	LILRB2	RIFIN		Modulate immune response for immune evasion	66
*Toxoplasma gondii*	LILRB1	HLA-G	NK cell	NA	277
	LILRB4		MDSC	Alter ARG1 and IL-10 expression via SHP2/STAT6, leading to MDSC dysfunction	278
*Trypanosoma cruzi*	LILRB1		CD4+ T cell	Modulate parasite-specific T cell response	279
Autoimmune diseases					
Autoimmune thyroid diseases	LILRB1		CD4+/CD8+ T cell	Disrupt IL-10 synthesis and cell proliferation	281
Rheumatoid arthritis	LILRB1	HLA-G	CD8+ T cell	Regulate CD8+ T cell activation	283
		Soluble HLA-G		Binding capacity dictates anti-inflammatory protection	284
Systemic lupus erythematosus	LILRB1		Peripheral blood B cell	Impaired LILRB1 expression and function	285
	LILRB4		Monocytoid DC	Loss-of-function polymorphisms affect inflammatory cytokine levels	106
	LILRB4		Plasmablast and plasma cell	Ectopically expressed	286
Multiple sclerosis	LILRB1	HLA-G		Modulate T-cell infiltration and immune-inflammatory response	287
	LILRB4			Influence Th1/Th17 proliferation and proinflammatory factor release	289
Neurodegenerative disease					
Alzheimer's disease	LILRB2/PirB	Aβ oligomers		Causes synaptic toxicity and impaired developmental plasticity	291
	LILRB2	Aβ oligomers, L-α-phosphatidylserine	Microglia	Inhibits TREM2 signaling	292
	LILRB4	ApoE	Microglia	Dampening microglia phagocytosis of amyloid plaques	84

Aβ, β-amyloid; ApoE, apolipoprotein E; ARG, Arginase-1; CHB, chronic hepatitis B; CMV, cytomegalovirus; DC, dendritic cell; DENV, dengue virus; EBV, Epstein-Barr virus; HIV-1, human immunodeficiency virus type 1; HLA, human leukocyte antigen; IFN-α, interferon-alpha; IL-10, interleukin-10; MDSC, myeloid-derived suppressor cell; NK, natural killer; RIFINs, repetitive interspersed families of polypeptides; S100A9, S100 calcium binding protein A9; SARS-CoV-2, severe acute respiratory syndrome coronavirus 2; TLR, toll-like receptor.

**Table 2:** LILRB expression and function in human malignancies.

Cancer types	LILRBs	Expression*	Clinical features	Survival (Prognosis)	Cellular roles	References
Hematological malignancies						
AML	LILRB1	Upregulated		OS (Poor)		210
	LILRB2	Upregulated		OS (Poor)	Support HSC expansion	72, 210, 211, 212, 293
	LILRB3	Upregulated		OS (Poor)	Enhance tumor cell survival, inhibit T cell activity	198, 210, 212, 233
	LILRB4	Upregulated		OS (Poor)	Support tumor cell infiltration and suppress T cell activity	22, 210, 212
	LILRB5	NS		OS (Favorable)		97, 212
CMML	LILRB4	Upregulated				213
MM	LILRB1	(a) Downregulated; (b) Upregulated	Cytogenetic abnormality t(4;14) translocation	OS (Poor)	Increase susceptibility to T/NK-mediated killing; Regulate cholesterol metabolism and protect tumor cells from ferroptosis	214, 297
	LILRB2	Downregulated				214
	LILRB3	Downregulated				214
	LILRB4	(a) NS; (b) Upregulated	Bone damage	OS (Poor)	Support tumor cell proliferation; Promote osteolytic lesions	116, 214, 215, 232, 299
	LILRB5	NS				214
CLL	LILRB1	Downregulated				216
	LILRB2	54.5% expression				300
	LILRB4	48.9% expression	Lymphoid tissue involvement		Regulate tumor progression	300, 301
ALL	LILRB2	Downregulated				302
KMT2A-rearranged ALL	LILRB4	Upregulated				217
ATL	LILRB4				Inhibit tumor cell growth	89
CTCL	LILRB1	100% expression			Inhibit tumor cell proliferation	303, 304, 305
cHL	LILRB2	Upregulated		OS (Poor)		309
Digestive system						
EC	LILRB1	Upregulated				221
	LILRB2	Upregulated	Tumor stage			312
HCC	LILRB1	Downregulated	TILs	RFS/PFS (Favorable)		313
	LILRB2	(a) Upregulated;(b) Downregulated	Gender, tumor size, cell differentiation, TILs	(a) OS (Poor); (b) OS/RFS/PFS/DFS (Favorable)	Promote macrophage polarization to M2 phenotype and recruit immunosuppressive T cells	196, 313, 314
	LILRB3	Downregulated	TILs	RFS/PFS (Favorable)		313
	LILRB4	Upregulated	TILs	RFS/PFS (Favorable)		313
	LILRB5	Downregulated	TILs	OS/RFS/PFS/DFS (Favorable)		313
GC	LILRB1	Upregulated	Pathological stage, cell differentiation, tumor size	OS (Poor)	Inhibit the anti-tumor effect of NK cells	205, 219, 220
	LILRB2	Upregulated				317
	LILRB4	Upregulated				220
PDAC	LILRB1	Upregulated; Downregulated	Pathological stage	OS (Poor)		219, 321
	LILRB2	Upregulated; Downregulated			Sustain EMT and the early metastatic behavior of tumor cells	319, 321
	LILRB3	Downregulated				321
	LILRB4	Downregulated		OS/RFS (Favorable)		321
CRC	LILRB1	Upregulated		OS (Poor)		219
	LILRB2	Upregulated	Gender, cell differentiation, vascular involvement, LN metastasis, tumor stage	OS (Poor)	Regulate tumor cell proliferation, invasion, migration, and angiogenesis	218, 231, 322, 323
	LILRB3	Upregulated	LN metastasis, tumor stage	OS/PFS (Poor)	Inhibit T-cell infiltration and promote M2-like TAM accumulation	324
	LILRB4	Upregulated	LN metastasis, tumor stage, CD45RO+ T cell count	OS (Poor)		325
Respiratory system						
NSCLC	LILRB1	51.5% expression	Tumor stage			107
	LILRB2	Upregulated	Cell differentiation, LN metastasis, tumor stage, age, TILs	OS/PFS (Poor)	Promote tumor growth, invasion, migration, and angiogenesis; Recruit M2-like TAMs and impair T cell response; Enhance resistance to radiation	197, 199, 222, 223, 224, 327, 328, 329
	LILRB4	Upregulated	Cell differentiation, tumor size, vascular involvement, tumor stage	OS (Poor)	Enhance tumor cell migration, invasion, and angiogenesis	230
Reproductive system						
BC	LILRB1	Upregulated	Pathological stage	OS (Poor)		219
	LILRB2	Upregulated	TILs, LN metastasis	OS/PFS (Poor)	Promote immune evasion; Promote tumor growth, and induce effector T cell senescence; Reprogram toward aerobic glycolysis	195, 229, 336, 337
OC	LILRB1	Upregulated	Age, tumor stage			225
Endometrial cancer	LILRB2	Upregulated		OS (Poor)	Support tumor cell expansion and migration	228
Urinary system						
ccRCC	LILRB1	60% expression				227
	LILRB2	60% expression				227
	LILRB3	Upregulated		OS (Poor)		344
Nervous system						
Glioma	LILRB1	Upregulated	Tumor size	OS (Poor)	Enhance tumor cell proliferation, migration and invasion	118
Glioblastoma	LILRB1	Upregulated				349
	LILRB2	Upregulated	Pathological stage	OS/DFS (Poor)	Induce MDSC formation and expansion	349, 350
	LILRB3	Upregulated				349
	LILRB4	Upregulated				349
Cutaneous tumors						
Melanoma	LILRB2	Upregulated			Promote tumor growth; Induce effector T cell senescence	229
Tumors of other system						
OSCC	LILRB1	Downregulated				352
TC	LILRB1	Upregulated	Pathological stage	OS (Poor)		219

ALL, acute lymphoblastic leukemia; AML, acute myeloid leukemia; ATL, acute T cell leukemia; BC, breast cancer; ccRCC, clear cell renal cell carcinoma; cHL, classical Hodgkin lymphoma; CLL, chronic lymphocytic leukemia; CMML, chronic myelomonocytic leukemia; CRC, colorectal cancer; CTCL, cutaneous T-cell lymphoma; DFS, disease-free survival; EC, esophageal carcinoma; EMT, epithelial-mesenchymal transition; GC, gastric cancer; HCC, hepatocellular carcinoma; HSC, hematopoietic stem cell; LN, lymph node; MDS, myelodysplastic syndrome; MDSC, myeloid-derived suppressor cell; MM, multiple myeloma; NS, no significance; NSCLC, non-small cell lung cancer; OC, ovarian cancer; OS, overall survival; OSCC, oral squamous cell carcinoma; PDAC, pancreatic ductal adenocarcinoma; PFS, progression-free survival; RFS, recurrence-free survival; TAMs, tumor-associated macrophages; TC, thyroid cancer; TILs, tumor-infiltrating lymphocytes; VEGF-C, vascular endothelial growth factor C. *Compare the expression levels of LILRBs between tumor cells and normal cells.

**Table 3:** Clinical trials targeting LILRB family in hematological malignancies and solid tumors.

Target	Agent	Drug type	Disease indication	Therapy	Intervention	Trial number	Phase and status	Trial duration
Hematological malignancies								
LILRB4	IO-202	IgG1 mAb	M-AML, CMML	Monotherapy and combination therapy	IO-202 alone; IO-202 and azacitidine (chemotherapy); IO-202 and azacitidine (chemotherapy) + venetoclax (BCL-2 inhibitor)	NCT04372433	Phase I (Completed)	Sep 2020 - Jan 2025
	LILRB4 CAR T	CAR T (Specific effector T-cells)	AML (M4/M5)	Monotherapy	Anti-LILRB4 CAR T cells	NCT04803929	Phase I (Recruiting)	Mar 2021 - Mar 2026
	LILRB4 STAR‑T	STAR-T (Specific effector T-cells)	AML	Monotherapy	LILRB4 STAR-T cells	NCT05518357	Phase I (Completed)	Jun 2022 - Nov 2022
	LILRB4 STAR-T	STAR-T (Specific effector T-cells)	AML	Monotherapy	LILRB4 STAR-T cells	NCT05548088	Phase I (Completed)	May 2023 - Aug 2024
	LILRB4 STAR-T	STAR-T (Specific effector T-cells)	AML, CMML	Monotherapy	LILRB4 STAR-T cells	NCT05739409	NA (Unknown status)	Feb 2023 - Aug 2024
Solid tumors								
LILRB1	BND-22/SAR444881	IgG4 mAb	Advanced solid tumors: BC, CC, CRC, EC, GC, HNSCC, HCC, gallbladder cancer, CCA, NSCLC, RCC, CSCC, PC, OC, and urothelial carcinoma	Monotherapy and combination therapy	BND-22/SAR444881 alone; BND-22/SAR444881 + pembrolizumab (anti-PD-1); BND-22/SAR444881 + cetuximab (anti-EGFR)	NCT04717375	Phase I/II (Active, not recruiting)	Apr 2021 - Jun 2025
	AGEN1571	IgG4 mAb	Advanced solid tumors	Monotherapy and combination therapy	AGEN1571 alone;AGEN1571 + balstilimab (anti-PD-1);AGEN1571 + botensilimab (anti-CTLA-4);AGEN1571 + balstilimab (anti-PD-1) + botensilimab (anti-CTLA-4)	NCT05377528	Phase I (Completed)	Jul 2022 - Dec 2024
	ADA-011	mAb	Advanced solid tumors	Monotherapy and combination therapy	ADA-011 alone; ADA-011 + PD-L1 inhibitor	NCT05601219	Phase I (Terminated)	Nov 2022 - Oct 2024
LILRB2	JTX-8064	IgG4 mAb	Advanced solid tumors: ccRCC, TNBC, OC, HNSCC, NSCLC, CSCC, UPS, liposarcoma, BTC	Monotherapy and combination therapy	JTX-8064 alone;JTX-8064 + pembrolizumab (anti-PD-1)	NCT04669899	Phase I/II (Completed)	Jan 2021 - Nov 2023
	IO-108	IgG4 mAb	Advanced solid tumors	Monotherapy and combination therapy	IO-108 alone; IO-108 + pembrolizumab (anti-PD-1);IO-108 + cemiplimab (anti-PD-1);	NCT05054348	Phase Ib (Completed)	Sep 2021 - May 2024
	MK-4830	IgG4 mAb	Advanced solid tumors: PC, glioblastoma, NSCLC, HNSCC, CCRCC, GC, OC, FTC, PPC, BC, mesothelioma	Monotherapy and combination therapy	MK-4830 alone; MK-4830 + pembrolizumab (anti-PD-1); MK-4830 + pembrolizumab (anti-PD-1) + lenvatinib (tyrosine kinase inhibitor), carboplatin, pemetrexed, paclitaxel and cisplatin (chemotherapies)	NCT03564691	Phase I (Active, not recruiting)	Jul 2018 - Sep 2025
	MK-4830	IgG4 mAb	High-grade serous OC	Combination therapy	MK-4830 + pembrolizumab (anti-PD-1) + chemotherapy	NCT05446870	Phase II (Completed)	Jul 2022 - Oct 2024
	ES009	IgG4 mAb	Advanced solid tumors	Monotherapy	ES009 alone	NCT06007482	Phase I (Completed)	Sep 2023 - Feb 2025
LILRB4	IO-202	IgG1 mAb	Advanced solid tumors	Monotherapy and combination therapy	IO-202 alone;IO-202 + pembrolizumab (anti-PD-1); RP2D of IO-202 + pembrolizumab (anti-PD-1)	NCT05309187	Phase I (Completed)	Apr 2022 - May 2024
	NGM831	IgG1 mAb	Advanced solid tumors: PC, BC, GC, NSCLC, CC, endocervical cancer, HNSCC, BUC, CRC, EC, OC, RCC, PCa, Melanoma, Mesothelioma, CCA	Monotherapy and combination therapy	NGM831; NGM831 + pembrolizumab (anti-PD-1); NGM831 + NGM438 (anti-LAIR1) + pembrolizumab (anti-PD-1)	NCT05215574	Phase I (Active, not recruiting)	Mar 2022 - Mar 2026
	MK-0482	IgG4 mAb	Advanced solid tumors	Monotherapy and combination therapy	MK-0482 alone; MK-0482 + pembrolizumab (anti-PD-1); MK-0482 + pembrolizumab (anti-PD-1) + paclitaxel, nab-paclitaxel, gemcitabine, carboplatin and pemetrexed (chemotherapies)	NCT03918278	Phase I (Completed)	Jun 2019 - Jun 2025
	MK-0482	IgG4 mAb	NSCLC	Combination therapy	MK-4830 + pembrolizumab (anti-PD-1)	NCT04165096	Phase II (Completed)	Jan 2020 - May 2025
LILRB1 and LILRB2	NGM707	BsAb (mAb)	Advanced solid tumors: Mesothelioma, glioblastoma, RCC, NSCLC, Melanoma, PC, GC, HNSCC, CCA, endocervical cancer, BC, OC, CC, CRC, EC	Monotherapy and combination therapy	NGM707 alone; NGM707 + pembrolizumab (anti-PD-1)	NCT04913337	Phase I/II (Active, not recruiting)	Jun 2021 - Jul 2025
LILRB2 and PD-1	CDX-585	BsAb (IgG-scFv)	Advanced solid tumors: NSCLC, GC, HNSCC, OC, PPC, FTC, BUC, CRC, EC, HCC, RCC, CCA, PC	Monotherapy	CDX-585 alone	NCT05788484	Phase I (Completed)	May 2023 - May 2025
LILRB1, LILRB2 and KIR3DL1	IOS-1002	TsAb (HLA-B57- B2m-IgG4_Fc fusion protein)	Advanced solid tumors	Monotherapy and combination therapy	IOS-1002 alone; IOS-1002 + pembrolizumab (anti-PD-1)	NCT05763004	Phase Ia/Ib (Recruiting)	Mar 2023 - May 2025

AML, acute myeloid leukemia; BC, breast cancer; BCL-2, B-cell lymphoma 2; BsAb, bispecific antibody; BTC, biliary tract cancer; BUC, bladder urothelial cancer; CAR T, chimeric antigen receptor T-cell; CC, cervical cancer; CCA, cholangiocarcinoma; ccRCC, clear cell renal cell carcinoma; CMML, chronic myelomonocytic leukemia; CRC, colorectal cancer; CSCC, cutaneous squamous cell carcinoma; CTLA-4, cytotoxic T-lymphocyte antigen 4; EC, esophageal carcinoma; EGFR, epidermal growth factor receptor; FTC, fallopian tube cancer; GC, gastric cancer; HCC, hepatocellular carcinoma; HNSCC, head and neck squamous cell carcinoma; IgG-scFv, immunoglobulin G single-chain variable fragment; LAIR1, leukocyte-associated immunoglobulin-like receptor 1; mAb, monoclonal antibody; M-AML, acute myeloid leukemia with monocytic differentiation; NA, not available; NSCLC, non-small cell lung cancer; OC, ovarian cancer; PC, pancreatic cancer; PCa, prostate cancer; PD-1, programmed cell death protein 1; PD-L1, programmed death ligand 1; PPC, primary peritoneal carcinoma; RCC, renal cell carcinoma; STAR‑T, synthetic T-cell receptor and antigen receptor; TNBC, triple-negative breast cancer; TsAb, trispecific antibody; UPS, undifferentiated pleomorphic sarcoma.

## Abbreviations

AD: Alzheimer's disease
AITD: autoimmune thyroid disease
ALCAM: activated leukocyte cell adhesion molecule
ALL: acute lymphoblastic leukemia
AML: acute myeloid leukemia
ANGPTL: angiopoietin-like protein
APC: antigen-presenting cell
ApoE: apolipoprotein E
ARG1: arginase-1
ATL: acute T-cell leukemia
Aβ: β-amyloid
BC: breast cancer
BCR: B-cell receptor
BM: bone marrow
CaMK: calcium/calmodulin-dependent protein kinase
CAR: chimeric antigen receptor
ccRCC: clear cell renal cell carcinoma
cFLIP: cellular FLICE-inhibitory protein
CLL: chronic lymphocytic leukemia
CMML: chronic myelomonocytic leukemia
CMV: cytomegalovirus
CNS: central nervous system
CRC: colorectal cancer
CREB: cAMP response element-binding protein
CTCL: cutaneous T-cell lymphoma
CTLA-4: cytotoxic T-lymphocyte antigen 4
CTL: cytotoxic T lymphocyte
DC: dendritic cell
DENV: Dengue virus
EBV: Epstein-Barr virus
ERK: extracellular signal-regulated kinase
F-actin: filamentous actin
FDA: food and drug administration
FcγR: Fc-gamma receptor
FGF-1: fibroblast growth factor 1
FTO: fat mass and obesity-associated protein
GA: glatiramer acetate
GC: gastric cancer
GLUT3: glucose transporter 3
HCC: hepatocellular carcinoma
HIV-1: Human Immunodeficiency Virus Type 1
HLA: human leukocyte antigen
HLA-I: HLA class I
HSC: hematopoietic stem cell
ICI: immune checkpoint inhibitor
IDO: indoleamine 2, 3-dioxygenase
IFN: interferon
Ig-like: immunoglobulin-like
IL-10: interleukin-10
IMC: immature myeloid cell
ITAMs: immunoreceptor tyrosine-based activation motifs
ITIMs: immunoreceptor tyrosine-based inhibitory motifs
JNK: c-Jun N-terminal kinase
KIRs: killer-cell immunoglobulin-like receptors
LDL: low-density lipoprotein
LDLR: LDL receptor
LDLRAP1: LDL receptor adapter protein 1
LILRBs: leukocyte immunoglobulin-like receptors, subfamily B
LN: lymph node
LPS: lipopolysaccharide
Lyn: Lck/Yes-related novel protein tyrosine kinase
MCC: merkel cell carcinoma
M.tuberculosis: Mycobacterium tuberculosis
m6A: N6-methyladenosine
mAbs: monoclonal antibodies
M-AML: monocytic differentiation-associated AML
MAPK: mitogen-activated protein kinase
MARCH9: membrane-associated RING-CH finger protein 9
mDCs: myeloid DCs
MDS: myelodysplastic syndrome
MDSC: myeloid-derived suppressor cell
MGUS: monoclonal gammopathy of undetermined significance
MHC-I: Major Histocompatibility Complex class I
MM: multiple myeloma
MS: multiple sclerosis
MTOC: microtubule organizing center
NASH: non-alcoholic steatohepatitis
NK: natural killer
NMIBC: non-muscle invasive bladder cancer
NSCLC: non-small cell lung cancer
OS: overall survival
OSCC: oral squamous cell carcinoma
*P.falciparum*: *Plasmodium falciparum*
PBMC: peripheral blood mononuclear cell
PC: prostate cancer
PDAC: pancreatic ductal adenocarcinoma
PD-L1: programmed cell death ligand 1
PI3K: phosphatidylinositol-4-phosphate 3-kinase
PirB: paired immunoglobulin-like receptor B
PKM2: pyruvate kinase muscle 2
PLC-γ: phospholipase C gamma
RA: rheumatoid arthritis
RFS: relapse-free survival
RIFINs: repetitive interspersed families of polypeptides
ROCK2: Rho-associated protein kinase 2
ROS: reactive oxygen species
S100A8: S100 calcium binding protein A8
S100A9: S100 calcium binding protein A9
SARS-CoV-2: severe acute respiratory syndrome coronavirus 2
Sema4A: semaphorin-4A
SHIP1: Src homology 2 domain-containing inositol phosphatase 1
sHLA-G: soluble HLA-G
SHP1/2: Src homology 2 domain-containing phosphatase 1/2
SIV: simian immunodeficiency virus
SLE: systemic lupus erythematosus
SNP: single nucleotide polymorphism
STAR: synthetic T-cell receptor and antigen receptor
Syk: Src family kinases: spleen tyrosine kinase
tDCs: tolerogenic DCs
*T.gondii*: *Toxoplasma gondii*
TAMs: tumor-associated macrophages
Th1: T helper 1
Th2: T helper 2
TILs: tumor-infiltrating lymphocytes
TLR: Toll-like receptor
TME: tumor microenvironment
TNF: tumor necrosis factor
Tr1: type 1 T regulatory
TRAF2: TNF receptor-associated factor 2
Treg: regulatory T cell
UPAR: urokinase plasminogen activator receptor
Vav1: Vav guanine nucleotide exchange factor 1
VEGF: vascular endothelial growth factor
ZAP70: zeta-chain-associated protein kinase 70 kDa
β2m: β2-microglobulin
